# Bayesian spatiotemporal forecasting and mapping of COVID‐19 risk with application to West Java Province, Indonesia

**DOI:** 10.1111/jors.12533

**Published:** 2021-05-07

**Authors:** I. Gede Nyoman M. Jaya, Henk Folmer

**Affiliations:** ^1^ Department of Economic Geography, Faculty of Spatial Sciences Groningen University Groningen The Netherlands; ^2^ Department of Statistics Padjadjaran University Bandung Indonesia

**Keywords:** Bayesian analysis, COVID‐19, forecasting, hotspot, mapping, pure model, spatiotemporal distribution

## Abstract

The coronavirus disease (COVID‐19) has spread rapidly to multiple countries including Indonesia. Mapping its spatiotemporal pattern and forecasting (small area) outbreaks are crucial for containment and mitigation strategies. Hence, we introduce a parsimonious space–time model of new infections that yields accurate forecasts but only requires information regarding the number of incidences and population size per geographical unit and time period. Model parsimony is important because of limited knowledge regarding the causes of COVID‐19 and the need for rapid action to control outbreaks. We outline the basics of Bayesian estimation, forecasting, and mapping, in particular for the identification of hotspots. The methodology is applied to county‐level data of West Java Province, Indonesia.

## 1 INTRODUCTION

The COVID‐19 pandemic has spread rapidly from Wuhan city, China, to at least 213 countries including Indonesia (Worldometer, 2020; Zhu et al., 2020). As of July 09, 2020, 70,736 cases were officially confirmed in Indonesia including 2657 deaths (Worldometer, 2020). A total of 3376 incidences and 186 deaths (6.85 incidences per 100,000 inhabitants with mortality rate of 5.51%) were confirmed in the 27 counties in West Java, Indonesia, on July 09, 2020 (COVID‐19 Information Center West Java, 2020).

COVID‐19 is primarily transmitted via the interaction of noncontaminated people with infected people through respiratory droplets or via contact with contaminated objects and surfaces (Liu et al., 2020). Close interaction in densely populated areas such as urban areas is more likely than in less populated areas, for example in rural areas. In a similar vein, people living in large households have a relatively high chance of infection because of more interaction among household members and because each family member may bring the virus home (Saadat et al., 2020). Social distancing, on the other hand, has a negative impact on the spread of the disease. Accordingly, the World Health Organization (WHO) recommends social distancing as the main approach to restrict transmission (WHO, 2020). Hence, restrictions on mobility have been imposed worldwide to mitigate the outbreak of COVID‐19 (Jiang & Luo, 2020; Rocklöv & Sjödin, 2020).

COVID‐19 varies geographically and temporally (Briz‐Redón & Serrano‐Aroca, 2020; Liu et al., 2020). To effectively prevent and control COVID‐19 transmission, it is crucial to map its spatiotemporal pattern and evolution. In addition, its present and future spatiotemporal distribution must be understood for etiological hypothesis generation and testing, healthcare planning, assessing policy performance, and optimally targeting limited resources (Jacquez, 2000; Jaya & Folmer, 2020a; Yang et al., 2020; Yin et al., 2014). This applies to all types of spatial levels, ranging from local, regional, national, to international. One challenge of modeling and mapping, especially at a fine spatiotemporal scale, is that the number of observations per geographical unit and time interval tends to be sparse. Hence, the majority of the geographical information systems and publications of COVID‐19 used cumulative incidences (Boulos & Geraghty, 2020; Roosa et al., 2020). However, that measure is confounded by differences in the underlying population, such as the population size or demographic structure, which tend to vary over space and time (Jamil et al., 2020; Mollalo et al., 2020). Accordingly, these measures result in biased mapping and predictions, notably overestimation (Kang et al., 2020). This problem can be solved using the standardized incidence ratio (SIR), which is defined as the ratio of the observed to the expected numbers of incidences for the population at risk (King et al., 2015). However, the SIR can become unreliable and result in the misconception of the true disease risk because of high sampling variability and spatiotemporal heterogeneity (Clayton & Kaldor, 1987; Yin et al., 2014). This problem can be solved by smoothing the SIR by accounting for spatiotemporal dependence and heterogeneity in estimating the relative risk (Anselin et al., 2006; Cressie, 1995).

Among the many smoothing approaches, Bayesian methods are widely used (Best et al., 2005). In the Bayesian framework, parameter estimates, inference, and prediction are based on the posterior distribution (Lawson, 2018) which expresses the probability of the parameters given the data. The posterior distribution is obtained by integrating (i) the prior distribution of the parameters according to historical or other kinds of external information, and (ii) the current sample via the likelihood function (Blangiardo & Cameletti, 2015). In contrast, frequentist approaches such as maximum likelihood derive parameter estimates from the likelihood only. Hence, Bayesian approaches allow evaluating uncertainty via two channels: likelihood and prior information. Estimates and predictions thus reflect the latest information via updating according to data collection and the latest prior information. Frequent updating of the uncertainty is important to modeling COVID‐19 because of the rapid changes of COVID‐19 incidence (Naumova, 2020; Williamson et al., 2020).

The Bayesian approach allows fitting of complex hierarchical models, taking into account a much wider class of conceptual models than non‐Bayesian approaches. In addition, it relies on relatively few assumptions (Dunson, 2001; Goicoa et al., 2012). Over the past few decades, the Bayesian approach to model spatial, temporal and spatiotemporal data has played an important role in numerous fields, including in epidemiological literature (Goicoa et al., 2012). It is commonly used to model spatiotemporal dependence and heterogeneity by random effects using a hierarchical structure on the parameters. Furthermore, it exploits the posterior distribution of the relative risk to smooth estimates (Blangiardo et al., 2013).

A problem in COVID‐19 modeling and mapping at all spatiotemporal scales is the insufficient knowledge regarding the causes of the transmission underlying human interaction and contact with contaminated objects (Saadat et al., 2020). Relevant risk factors are still emerging with great uncertainties, and many are difficult to measure (Subramanian et al., 2020). Hence, modeling COVID‐19 has proven to be a complex and difficult endeavor. There is growing evidence, however, that COVID‐19 affects different demographic and socioeconomic groups in distinct ways. Healthcare workers, older age groups, and people with poor health are more susceptible to infection and vulnerable to negative health outcomes than younger people and those with good health (Saadat et al., 2020; Williamson et al., 2020). Risk factors in particular include being over the age of 70 or having medical problems such as chronic respiratory diseases, diabetes, cardiovascular diseases, obesity, and cancer (Fang et al., 2020; Giannisa et al., 2020; Zheng et al., 2020). Socioeconomic factors such as occupation, education, lifestyle, and socioeconomic status have also been found to affect the COVID‐19 infection risk (Khalatbari‐Soltani et al., 2020; Stojkoski et al., 2020). Weather variables, such as temperature and humidity, and environmental conditions, notably air pollution, have been designated as risk factors that affect transmission (Ma et al., 2020). However, other studies reported contradictory results and claimed that the relevance of weather and environmental conditions as risk factors is questionable (Tosepu et al., 2020; Xie & Zhu, 2020). Hence, a comprehensive overview of the determinants of the spatiotemporal distribution of COVID‐19 is still lacking. However, even if knowledge of the risk factors is available, it is debatable if updated data is readily available for estimation and prediction using complex models. According to M. Wang and Flessa (2020), the formulation and computation of even the best COVID‐19 models are under severe uncertainty. This applies particularly to developing countries such as Indonesia, where data availability and accuracy is a common issue (Djalante et al., 2020).

The impacts of the unobserved factors affecting COVID‐19 risk transmission can be inferred from changes in the number of incidences in space and time captured by hierarchical random effects statistical models, denoted as pure models (Lopez‐Quılez & Munoz, 2009). As in time series analysis, when there is lack of solid information on the covariates or uncertainty in the model specification, univariate, that is, pure models are commonly applied as alternatives to multivariate models to generate short‐term forecasts (Chayamaa & Hirata, 2016). Additionally, Martinez‐Beneito and Botella‐Rocamora (2019) observed that random effects models are a suitable tool for modeling correlated data in general. A spatiotemporal pure model consists of structured and unstructured spatial and temporal random effects and their interactions, thus capturing the unobserved factors driving the spatiotemporal distribution of the relative risk (Jaya & Folmer, 2020a; Kazembe, 2007; Wakefield, 2007). A hierarchical pure model provides robust estimation of the spatiotemporal relative risk parameters. However, care is required in interpreting the model, because it may not reflect any causal mechanism due to the fact that it does not contain explicit information about the risk factors (Wakefield, 2007). It is an exercise in mapping and forecasting in the first place. The spatiotemporal pure model introduced in this paper is a generalization of the pure time series model, although in a Bayesian setting. As its time series analog, it generates unbiased and precise forecasts with minimal data requirements. It is generally accepted that when the goals are mapping and forecasting, a simple model without exogenous variables suffices as a first approximation (Leone, 1979; Naylor et al., 1972; Preez & Witt, 2003). Even more so, the more parsimonious the model, the wider its applicability.

The pure model does not contain explicit information on the risk factors. It can, however, be used to produce maps to generate new hypotheses on the disease etiology, particularly to identify relevant risk factors (Huque et al., 2016; Jaya & Folmer, 2020a; Wakefield, 2007). Note that great care is required in modeling covariates at an early stage of research on an unexplored topic because of uncertainty about nonlinear or interacting effects of the covariates (Wakefield, 2007).

Rather than just observing relative risk estimates, it is important to define “unusual” counties where public intervention is needed (Aguayo et al., 2020). The unusual counties are denoted “hotspots,” that is, isolated areas in which the relative risk is unusually high. Bayesian methods are convenient to define hotspots via a classification decision rule. The rule defines a reference threshold on the posterior probability distribution of the relative risk, and a cutoff value on the posterior probability distribution that the relative risk is above the reference threshold. Then, a county is classified as a hotspot if the posterior probability that the relative risk is above the reference threshold is greater than the cut‐off value (Richardson et al., 2004). Hotspots can provide clues to disease etiology, suggesting, for example, local environmental or social characteristics that promote increased risk (Mclafferty, 2015).

Herein, we present a parsimonious county‐level space–time model and corresponding maps of the distribution of COVID‐19, outline the basics of Bayesian mapping and forecasting, and apply it to West Java Province, Indonesia, as an illustration. As a first step, we estimated several Bayesian spatiotemporal models and selected the most appropriate one using Bayesian evaluation criteria. Next, we interpreted the posterior means (intercept, random effects) and standard errors which were used to plot maps for the relative risk by county and week. The maps were subsequently used to identify high‐risk counties (hotspots) using the selected exceedance probability.

The remainder of this paper is structured as follows. In Section 2 we present the spatiotemporal pure model to describe and forecast the relative risk distribution. Section 3 presents the estimations, forecasts, and maps of COVID‐19 relative risk for West Java, and Section 4 contains the discussion and conclusions. Supporting information on methods and material, including supplements on data and detailed relative risk estimates for each county, is presented in Appendices A and B.

## 2 THE RELATIVE RISK AS A PURE MODEL

The spatiotemporal pure model presented below only requires the number of incidences and population size per region and period. It assumes that the number of COVID‐19 incidences in county *i* in period *t*, $y_{\mathrm{it}}$, follows a Poisson distribution (or, in the case of overdispersion when the data contains a large number of zeros, a negative binomial (NB) distribution (Berk & MacDonald, 2008; Payne et al., 2017) or a zero‐inflated Poisson (ZIP) distribution (Lewsey & Thomson, 2004) or a zero‐inflated negative binomial (ZINB) distribution (Agarwal et al., 2002) as follows:

(1) $$y_{\mathrm{it}}|\lambda_{\mathrm{it}}\sim \text{Poisson}(\lambda_{\mathrm{it}}),$$

where $\lambda_{\mathrm{it}}$ denotes the mean and variance of $y_{\mathrm{it}}$. To capture differences in the population at risk, $\lambda_{\mathrm{it}}$ is divided by the expected number of incidences, $E_{\mathrm{it}}$ defined as follows (Jaya & Folmer, 2020a):

(2) $$E_{\mathrm{it}}=N_{\mathrm{it}}p,\;i=1,\ldots ,n\;\text{and}\;t=1,\ldots ,T,$$

where $N_{\mathrm{it}}$ is the population at risk, *n* the number of observed counties, *T* the number of observed periods, and *p* the constant disease probability across all regions, that is, $p=\frac{\left(\sum_{i=1}^{n}\sum_{t=1}^{T}y_{\mathrm{it}},)\right)/\mathrm{nT}}{(\overset{n}{\underset{i=1}{\sum }}\sum_{t=1}^{T}N_{\mathrm{it}})//\mathrm{nT}}$ (Abente et al., 2018; Jaya & Folmer, 2020a). Accordingly, $y_{\mathrm{it}}$ follows a Poisson distribution with the mean and variance equal to $\lambda_{\mathrm{it}}=E_{\mathrm{it}}\theta_{\mathrm{it}}$, as follows:

(3) $$y_{\mathrm{it}}|\lambda_{\mathrm{it}}\sim \text{Poisson}(E_{\mathrm{it}}\theta_{\mathrm{it}}).$$

The ratio of the observed number of incidences ($y_{\mathrm{it}}$) to the expected number of incidences ($E_{\mathrm{it}}$) is the SIR. It is the unbiased maximum likelihood estimator of the relative risk (Jaya et al., 2017) defined as

(4) $${\mathrm{SIR}}_{\mathrm{it}}={\hat{\theta }}_{\mathrm{it}}=\frac{y_{\mathrm{it}}}{E_{\mathrm{it}}},\;i=1,\ldots ,n\;\text{and}\;t=1,\ldots ,T.$$

Although the SIR is an unbiased estimator of the relative risk, it can be unreliable for sparse count data. Particularly, a spatiotemporal unit with a small number of observed incidences with a small population size, and hence a low expected number of cases, may be incorrectly classified as a high‐risk unit (Jaya & Folmer, 2020a; Yin et al., 2014). To reduce the amount of variation due to population size and heterogeneity, smoothing can be applied (Jaya et al., 2017). Spatiotemporal smoothing effectively borrows strength across spatiotemporal units by introducing spatiotemporal dependence and heterogeneity into the Poisson or NB regression model (Yin et al., 2014). Taking the natural logarithm of $\lambda_{\mathrm{it}}$ yields

(5) $$\log\left(\lambda_{\mathrm{it}}\right)=\log(\text{offset}(E_{\mathrm{it}}))+\log\left(\theta_{\mathrm{it}}\right).$$

The offset in Equation (5) is assumed to have a regression coefficient fixed at 1.

To analyze the spatiotemporal distribution of COVID‐19, we consider the loglinear relative risk, and model it as a generalized linear mixed model accounting for the spatiotemporal dependence and heterogeneity as follows (For inclusion of age‐structure effects, see Appendix A1):

(6) $$\eta_{\mathrm{it}}=\alpha +\omega_{i}+\upsilon_{i}+\phi_{t}+\varphi_{t}+\delta_{\mathrm{it}}.$$

Here, $\eta_{\mathrm{it}}=\log\left(\theta_{\mathrm{it}}\right),\alpha$ denotes the intercept representing the overall relative risk; $\omega_{i},\upsilon_{i},\phi_{t},$ and $\varphi_{t}$ are the spatially structured (spatial autocorrelation), spatially unstructured (spatial heterogeneity), temporally structured (serial autocorrelation), and temporally unstructured (temporal heterogeneity) random effects, respectively, and $\delta_{\mathrm{it}}$ is the interaction of a pair of the four random effects above. The random effects implicitly capture the determinants of the relative risk (Jaya & Folmer, 2020a; Kazembe, 2007).

We propose Bayesian inference to analyze model (6) which takes all the parameters as random variables with prior probability distributions. We propose a vague Gaussian prior distribution with a zero mean and a large variance for $\alpha :\alpha \sim N(0,10^{6})$ (Martinez‐Beneito & Botella‐Rocamora, 2019). In Bayesian (disease) mapping, spatial autocorrelation in the data is commonly modeled at the second level of a hierarchical model by a set of random effects with CAR prior distributions belonging to the class of Markov random fields. Besag et al. (1991) proposed the intrinsic and convolution CAR priors. However, these CAR priors do not allow clear distinguishing between the structured and unstructured random effects, which are thus not identifiable (MacNab, 2011). Leroux et al. (2000) proposed the Leroux before make the difference between the unstructured and structured spatial variation explicit. This prior is also more flexible in representing a range of spatial correlation scenarios (Lee, 2011). Through a simulation study, Aswi et al. (2020) found that spatial estimation based on the Leroux prior performed best on the Watanabe–Akaike information (WAIC) criterion. Krisztin et al. (2020) proposed to use spatial econometrics to quantify spatial spillover based on the SAR model (in spatial econometrics known as the spatial error model). Rather than using latent random effects as in the Bayesian approach, spatial econometrics models spatial dependence as an explicit spatial autoregressive error structure.

In addition to the LCAR prior proposed by Aswi et al. (2020), we also considered the CAR and SAR priors for the application in Section 3. The results presented in Table B4 show that the LCAR prior performed best. For the spatially structured random effect (ω) of county i across time t the LCAR prior reads as follows (Leroux et al., 2000):

(7) $$\omega_{i}|\omega_{-i},W\sim N\left(\frac{\rho \overset{n}{\underset{j=1}{\sum }}w_{\mathrm{ij}}\omega_{j}}{\rho \overset{n}{\underset{j=1}{\sum }}w_{\mathrm{ij}}+1-\rho },\frac{\sigma_{\omega }^{2}}{\left(\rho \overset{n}{\underset{j=1}{\sum }}w_{\mathrm{ij}}+1-\rho ,)\right)},)\right),\;\forall t,\;i=1,\ldots ,n,$$

where $W=(w_{\mathrm{ij}})$ is the spatial weights matrix, ρ is the spatial autoregressive parameter, and $\sigma_{\omega }^{2}$ the variance hyperparameter of $\omega =(\omega_{1},\ldots ,\omega_{n})\prime$.

Tsai et al. (2009) and Duncan et al. (2017) concluded that first‐order adjacency weights (with $w_{\mathrm{ij}}=1$ if i and j share a vertex or border, and $w_{\mathrm{ij}}=0$ otherwise) are generally an optimal choice for modeling spatial spillover and spatial smoothing. Nevertheless, we compared it with various types of weights matrices based on the geographical distance ${(d}_{\mathrm{ij}})$ between areas i and j measured as the Euclidean distance between their respective centroids. The results are presented in Table B5. Table B5 shows that there is no uniformly best model. However, the first‐order queen contiguity spatial weights matrix with ρ=0.347 is the best, or close to the best, weights matrix. Hence, we selected it for further analysis.

A vague Gaussian prior with large variance is assigned to log(ρ/(1−ρ))~N(0,10). (The transformation is used to ensure that ρ takes values between 0 and 1 [Bivand et al., 2015; Martinez‐Beneito & Botella‐Rocamora, 2019]). For the spatially unstructured random effect $v=(v_{1},\ldots ,v_{n})\prime$, we propose an exchangeable prior as follows (Jaya & Folmer, 2020a):

(8) $$\upsilon_{i}|\sigma_{\upsilon }^{2}\sim N\left(0,\sigma_{\upsilon }^{2},)\right),\;\forall t,\;i=1,\ldots ,n,$$

where $\sigma_{\upsilon }^{2}$ is the variance parameter of υ.

A random walk prior of order one (RW1)

(9) $$\phi_{t+1}-\phi_{t}|\sigma_{\phi }^{2}\sim N\left(0,\sigma_{\phi }^{2},)\right),\;\forall i,\;t=1,..,T,$$

or order two (RW2)

(10) $$\phi_{t}-2\phi_{t+1}+\phi_{t+2}|\sigma_{\phi }^{2}\sim N\left(0,\sigma_{\phi }^{2},)\right),\;\forall i,\;t=1,\ldots ,T,$$

is appropriate for the temporal trend $\phi =(\phi_{1},\ldots ,\phi_{T})\prime$ with $\sigma_{\phi }^{2}$ the variance hyperparameter of ϕ. For the unstructured temporal effects $\varphi =(\varphi_{1},\ldots ,\varphi_{T})\prime$ we propose the following exchangeable prior:

(11) $$\varphi_{t}|\sigma_{\varphi }^{2}\sim N\left((,0,\sigma_{\varphi }^{2},)\right),\;\forall i,\;t=1,\ldots ,T,$$

where $\sigma_{\varphi }^{2}$ is the variance hyperparameter of φ. For the four interaction components $\delta =(\delta_{11},\ldots ,\delta_{\mathrm{nT}})\prime$, the priors are the products of the priors of the corresponding components (see Boulos & Geraghty, 2020; Jaya & Folmer, 2020a; Knorr‐Held, 2000 for details).

In addition to the priors above, the variance hyperparameters $\psi =\left((,\sigma_{\omega }^{2},\sigma_{\upsilon }^{2},\sigma_{\phi }^{2},\sigma_{\varphi }^{2},\sigma_{\delta }^{2},)\right)\prime$ require priors (hyperpriors). A typical option is the non‐informative inverse gamma distribution (IG) with shape and scale parameters of values approximately zero. That is: $\{\sigma_{\omega }^{2},\sigma_{\upsilon }^{2},\sigma_{\phi }^{2},\sigma_{\varphi }^{2},\sigma_{\delta }^{2}\}$~ IG (0.001, 0.001). However, the IG prior does not have an appropriate limiting posterior distribution (Gelman, 2006). Consequently, posterior inferences are sensitive to the shape and scale parameters. To overcome this problem, the half‐Cauchy distribution with scale parameter 25 is an appropriate, weakly informative alternative hyperprior distribution for $\{\sigma_{\omega }^{2},\sigma_{\upsilon }^{2},\sigma_{\phi }^{2},\sigma_{\varphi }^{2},\sigma_{\delta }^{2}\}$ (Gelman, 2006).

Hence, it follows that the number of COVID‐19 incidences $y_{i}=(y_{i1},\ldots ,y_{\mathrm{iT}})\prime$ has unknown parameters $\Omega =(\alpha ,\omega^{\prime },\rho ,\upsilon \prime ,\phi \prime ,\varphi \prime ,\delta \prime )\prime$ defined as random variables with priors p(Ω|ψ) and hyperparameters $\psi =\left((,\sigma_{\omega }^{2},\sigma_{\upsilon }^{2},\sigma_{\phi }^{2},\sigma_{\varphi }^{2},\sigma_{\delta }^{2},)\right)\prime$ with hyperprior distributions p(ψ).

The Bayesian pure spatiotemporal Model (6) can be conveniently estimated via the integrated nested Laplace approximation procedure (INLA) (Rue et al., 2009) using the R‐INLA package (Blangiardo et al., 2013). For details of INLA, see the Supporting Information in Appendix A2. The posterior means of the parameters of Model (6), their standard deviations, forecasts, and some other statistics are computed based on their marginal posterior distributions (Gelman et al., 2013; Jaya & Folmer, 2020a; X. Wang et al., 2018). Furthermore, the INLA package yields the deviance information criterion (DIC) (Spiegelhalter et al., 2002) and Watanabe–Akaike information criterion (WAIC) (Watanabe, 2010) as goodness‐of‐fit statistics. INLA also yields the marginal predictive‐likelihood (MPL) (Dey et al., 1997), mean absolute error (MAE), root mean squared error (RMSE) (Pal, 2017), and Pearson correlation coefficient (*r*) (Santa et al., 2019), which are appropriate statistics for prediction performance evaluation as such (if all the observations are used to estimate the model), and for evaluation based on cross‐validation.

In a Bayesian setting, forecasts of the relative risk are based on the posterior predictive distribution $p(\hat{y}\mathrm{|y})$ (X. Wang et al., 2018). In INLA, prediction can be conveniently accomplished by fitting a model with missing observations. Specifically, one combines past observations from the previous periods and missing or not available (NA) observations for the periods one wants to forecast. For details on forecasting using INLA see Appendix A2. Choropleth maps are commonly used to visualize the spatiotemporal distribution of the relative risk of a disease (Indrayan & Kumar, 1996).

To identify spatiotemporal hotspots, we employed exceedance probability criteria (Lawson, 2010). The spatiotemporal exceedance probability can be computed from the posterior spatiotemporal distribution of the relative risk. It is defined as the probability that the estimated posterior mean of the relative risk of area i at time t is higher than a threshold value c, that is, $\text{Pr}(\theta_{\mathrm{it}}>\mathrm{c|}y).$ It is estimated as

(12) $$\hat{\text{Pr}}(\theta_{it}>c|y)=1-\int_{\theta_{it}\leq c}p(\theta_{it}|y)d\theta_{it},$$

where $\int_{\theta_{it}\leq c}p(\theta_{it}|y)d\theta_{it}$ is the cumulative probability of $\theta_{it}$ with threshold value c. It can be estimated using the Laplace approximation (Blangiardo & Cameletti, 2015). The use of the exceedance probability to identify hotspots requires two parameters that must be fixed a priori. The first one is the threshold value c for $\theta_{\mathrm{it}}$. The value 1 indicates that a county has average relative risk, whereas values such as 2 or 3 indicate extreme risk. The second parameter is the cut‐off value γ of the exceedance probability. Common values for γ are 0.90, 0.95 and 0.99 (Lawson & Rotejanaprasert, 2014). See Jaya and Folmer (2020b) for a detailed discussion of hotspots.

## 3 SPATIOTEMPORAL MAPPING AND FORECASTING OF COVID‐19 RELATIVE RISK IN WEST JAVA

The data were obtained from Statistics West Java (2019) and COVID‐19 Information Center West Java (2020), respectively. The following observations apply. First, the number of tests may not be constant overtime. Indeed, it increased in many, especially industrialized countries as the duration of the pandemic progressed (Papastefanopoulos et al., 2020; Subramanian et al., 2020). The number of confirmed COVID‐19 cases tends to increase with the number of tests (Bertozzi et al., 2020; Roda et al., 2020). However, the number of tests in West Java over the study period (March 06–July 09, 2020) was approximately constant at around 25,000 tests per week due to limited testing capacity (Nabila, 2020). Second, we did not take into account the age effect, although there is growing evidence that the relative risk and impacts including mortality vary by age (Goldstein et al., 2021; Mallapaty, 2020). Particularly, older people have a substantially larger chance of getting infected and developing symptoms (Mallapaty, 2020). We did not include the age effect because our data refers to the beginning of the pandemic in Indonesia, at which point incidences were not registered by age (Djalante et al., 2020).

**Figure 1:**
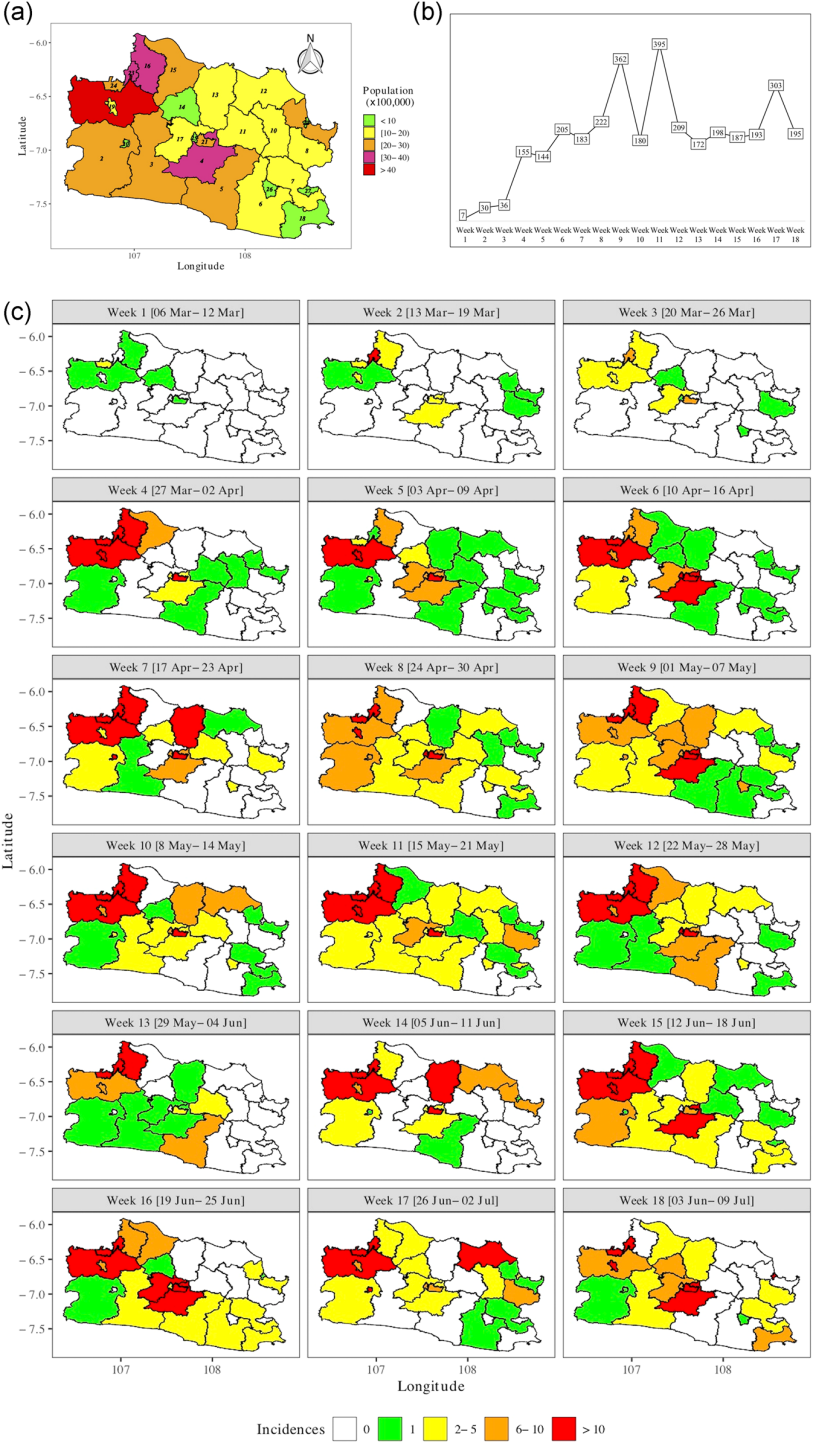
(a) Population at risk per county in 2019 (×100,000). (b) Weekly total number of confirmed COVID‐19 incidences March 06–July 09, 2020 and (c) weekly number of confirmed COVID‐19 incidences per county, March 06–July 09, 2020. The ids (italics) in (a) correspond to the *ids* in Table B1 [Color figure can be viewed at wileyonlinelibrary.com]

Figure 1a shows the population at risks by census tract in West Java in 2019. The most populous counties are in the North West and center. See Appendix B for details. Figure 1b depicts the weekly total number of confirmed COVID‐19 incidences March 06–July 09, 2020. Figure 1b shows a rapid increase in the number of confirmed incidences from Week 1 till Weeks 9–11 (with a dip in Week 10), followed by a substantial decline and leveling off from Week 12 till Week 18 with a short peak in Week 17. Figure 1c shows that the outbreak started in the populated counties in the northeast and center, and started rapidly spreading to their surrounding counties. From Week 12 onward, the disease started leveling off in the periphery whereas the populated counties and, to a lesser extent, their neighboring counties remained affected.

To identify the most appropriate spatiotemporal model of COVID‐19, we estimated 48 different submodels of Model (4) with the RW1 and RW2 trends for the Poisson and NB distributions and their zero‐inflated variants. We compared their fits using the DIC and WAIC, and evaluated their predictive performances using the MPL, MAE, RMSE, and *r* based on cross‐validation with the first 14 weeks as the training set and Weeks 15–18 as the testing set. The results are presented in Table B2.

Apart from a few minor exceptions, the evaluation statistics of the RW1 submodels outperformed their RW2 counterparts. Hence, the latter were not considered any further. Similarly, the ZIP and the ZINB distributions were rejected in favor of the Poisson and NB distribution, respectively, thereby reducing the selection of the optimal model to the six variants of the Poisson and NB models. As a next step, we assessed overdispersion by means of the deviance index of the Poisson models and the overdispersion parameter of the NB models (Mohebbi et al., 2014). A deviance index greater than one is evidence of overdispersion. Table B3 shows that the deviance index of the six Poisson models ranged from 1.49 to 2.47, and that the overdispersion parameters of the NB models were larger than zero. Therefore, the Poisson model was rejected in favor of the NB model.

Table B2 shows that Models M1 and M2 have larger fit indices (DIC and WAIC) than Models M3–M6, with an interaction term indicating that space–time interaction should be considered. Among the models with the interaction term, Model M4 has the largest MPL and *r* and the smallest MAE and RMSE values, indicating that Model M4 performs the best. Hence, it was selected for further analysis. The posterior means, standard errors, and credible intervals of the fixed and random effects of Model M4 are presented in Table 1.

**Table 1:** Posterior means, standard errors (*SE*), and credible intervals (CI) of the fixed and random effects of model M4

	Mean	*SE*	95% CI
Fixed effect			
Intercept	–0.449	0.241	(–0.922; –0.024)
Spatial autocorrelation (ρ)	0.347	0.302	(0.004; 0.948)
Random effects			
Structured spatial effect (${\hat{\sigma }}_{\omega }^{2}$)	0.152	0.276	(0.002; 0.815)
Unstructured spatial effect (${\hat{\sigma }}_{v}^{2}$)	0.076	0.111	(0.001; 0.361)
Structured temporal effect (${\hat{\sigma }}_{\phi }^{2}$)	0.777	0.517	(0.230; 2.113)
Unstructured temporal effect (${\hat{\sigma }}_{\varphi }^{2}$)	0.062	0.093	(0.001; 0.300)
Interaction effect (${\hat{\sigma }}_{\delta }^{2}$)	0.028	0.022	(0.003; 0.082)
Fraction of the variance (FV)			
${\text{FV}}_{{\hat{\sigma }}_{\omega }^{2}}$	0.139		
${\text{FV}}_{{\hat{\sigma }}_{v}^{2}}$	0.069		
${\text{FV}}_{{\hat{\sigma }}_{\phi }^{2}}$	0.710		
${\text{FV}}_{{\hat{\sigma }}_{\varphi }^{2}}$	0.057		
${\text{FV}}_{{\hat{\sigma }}_{\delta }^{2}}$	0.026		

Table 1 shows that the intercept *α* = −0.449 (95% credible interval [CI] [–0.922; –0.024]), which provides the mean relative risk as exp(−0.449) = 0.638. The posterior mean of the spatial dependence parameter ρof the LCAR prior is 0.347 (95% CI [0.002; 0.948]), indicating small and imprecise spatial transmission between neighboring counties. (We also considered the CAR and SAR prior and several distance‐based contiguity matrices as alternatives to the first‐order queen contiguity matrix. The results are presented in Table B4).

The contribution of each random effect and the interaction term to the total variance, FV_*h*_, is calculated as ${\text{FV}}_{h}={\hat{\sigma }}_{h}^{2}/\overset{H}{\underset{h=1}{\sum }}{\hat{\sigma }}_{h}^{2}\mathrm{;h}=1,\ldots ,5$ with ${\hat{\sigma }}_{h}^{2}=${${\hat{\sigma }}_{\omega }^{2},{\hat{\sigma }}_{\upsilon }^{2},{\hat{\sigma }}_{\phi }^{2},{\hat{\sigma }}_{\varphi }^{2},{\hat{\sigma }}_{\delta }^{2}$}. Table 1 shows that the structured spatial (ω) and unstructured spatial random effects (v) account for (0.139 + 0.069) × 100% = 20.8% of the spatiotemporal COVID‐19 variation, the structured (ϕ) and unstructured temporal effects (φ) for (0.710 + 0.057) × 100% = 76.6%, and the interaction effect (δ) for 2.6%. Accordingly, the temporal effects dominate the spatiotemporal variation of COVID‐19.

**Figure 2:**
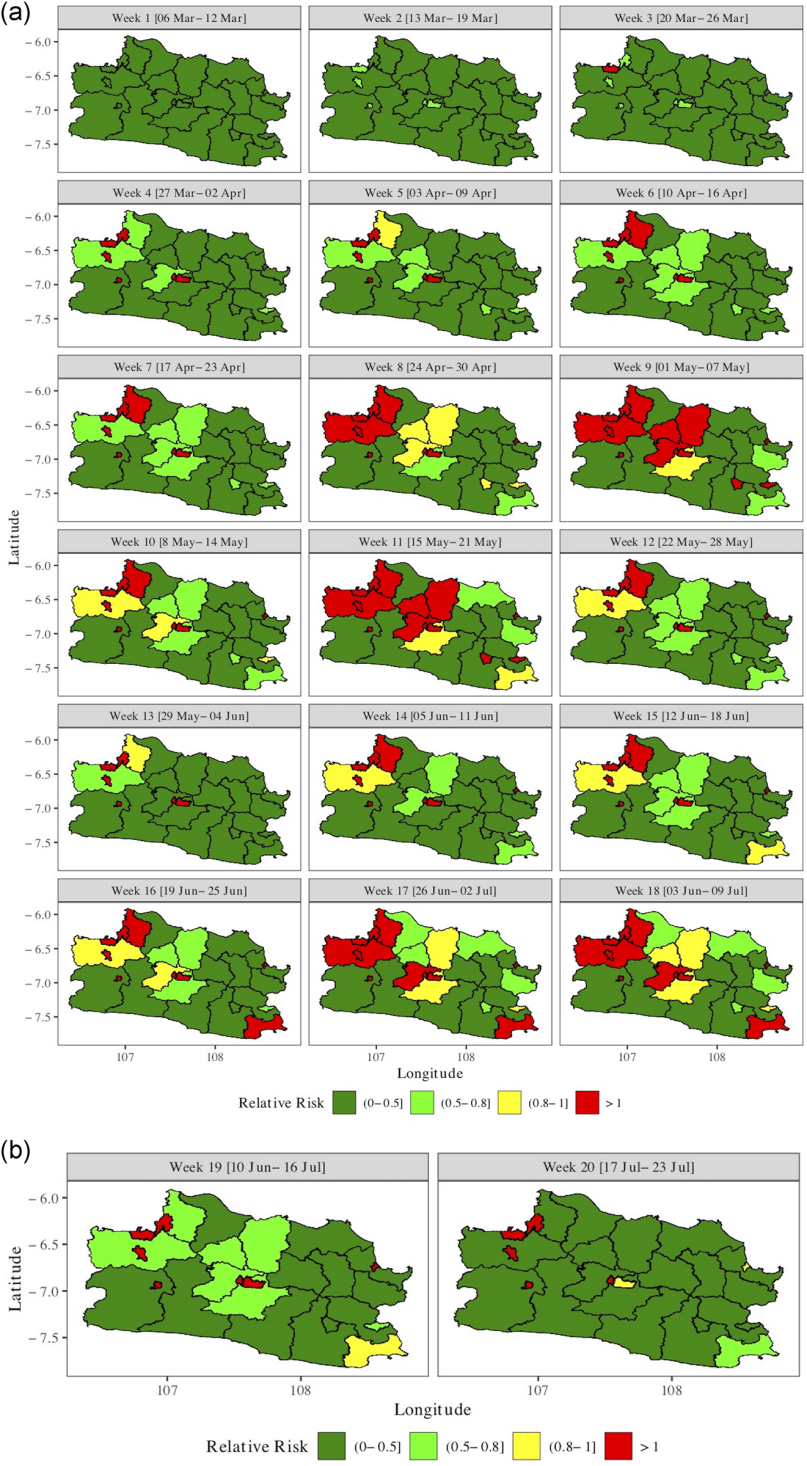
Choropleth maps of (a) estimated (Weeks 1–18) and (b) predicted (Weeks 19–20) relative risk [Color figure can be viewed at wileyonlinelibrary.com]

Figure 2 presents the choropleth maps of the estimated (Weeks 1–18) and predicted relative risk (Weeks 19 and 20) for the 27 counties based on the spatiotemporal model with parameters presented in Table 1. A relative risk larger than one means that the corresponding posterior mean is larger than average across space and time. Figure 2a shows that the relative risk in all counties was low during the first two weeks. It began at a relatively high intensity (relative risk >1, in red) in one county in the northwest in Week 3, then began spreading with light (relative risk 0.5–0.8, light green) to high intensity in the northwest, and emerged in the center in Week 4. During Weeks 5–12, it spread further and intensified in the latter regions while pockets emerged in the southwest. During Weeks 13–18, a substantial fluctuation occurred. Initially, a rapid decline was observed in the northwest and center, followed by a gradual increase in these areas for the remainder of the period, ranging from light to high intensities. Figure 2b shows that for Weeks 19 and 20, a significant decrease was predicted in these counties, except in the northwest counties and in the center where the outbreak started.

**Figure 3:**
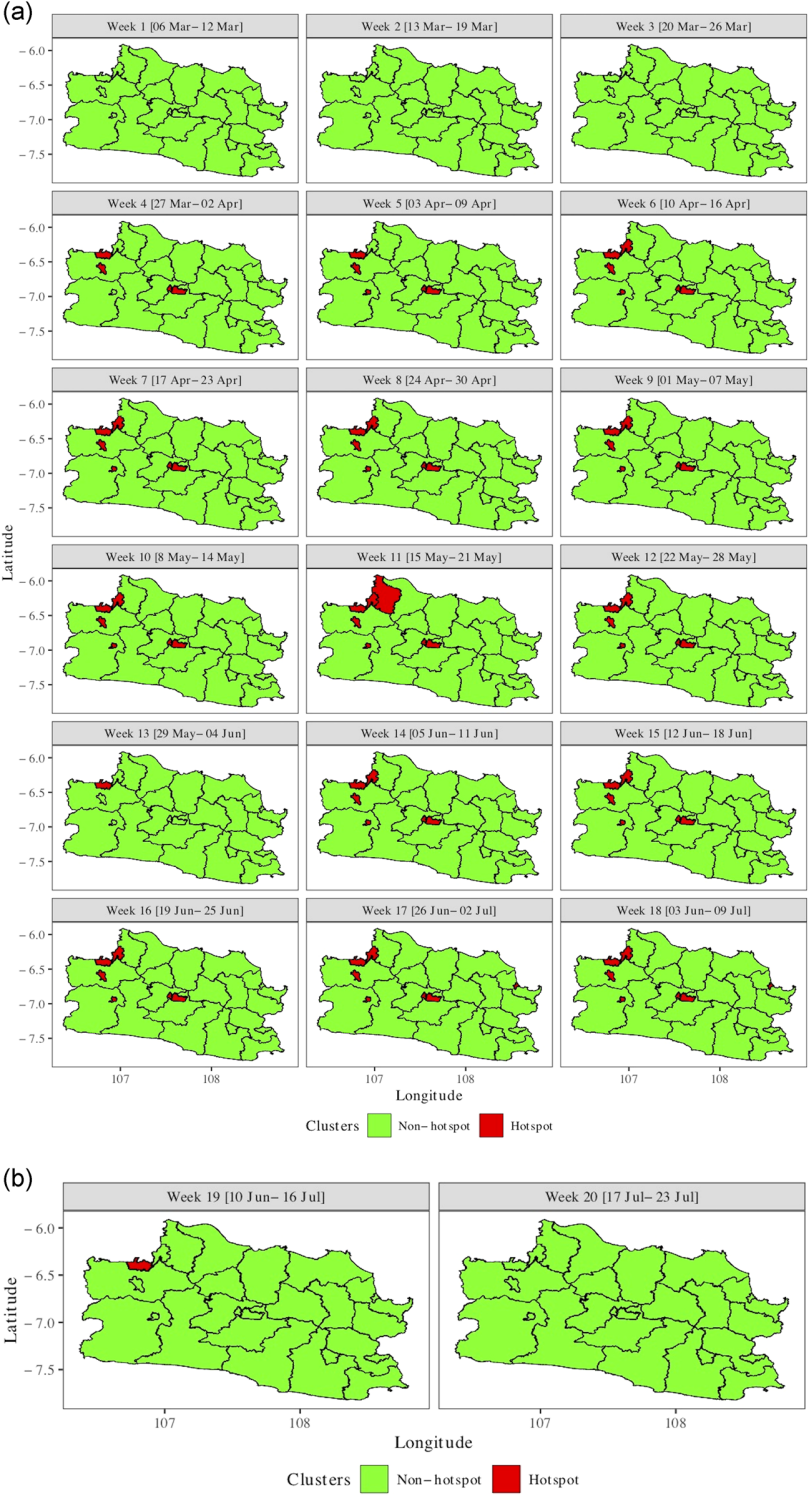
95% posterior exceedance probability of relative risk exceeding 1 ($\hat{\text{Pr}}(\theta_{\mathrm{it}}>1|y>0.95)$): (a) estimated (Weeks 1–18) and (b) predicted (Weeks 19–20) [Color figure can be viewed at wileyonlinelibrary.com]

In terms of policy, the identification of hotspots is crucial because, as epicenters, close monitoring and effective managing of them are required to prevent proliferation. Figure 3 presents hotspots with posterior exceedance probability *γ *= 95% ($\hat{\text{Pr}}(\theta_{\mathrm{it}}>1|y)>0.95$) for Weeks 1–18 and the predicted hotspots for Weeks 19 and 20. The figure shows that the first hotspots emerged in the northwest and the center in Week 4. The counties concerned remained as hotspots during the observation period. In addition, some northwest counties in the vicinity (not neighboring) of the original hotpots became and remained as hotpots. After a peak in Week 11, the number of hotspots started declining although the early hotspots remained through Week 18. For Weeks 19 and 20, a decline was predicted. First, the hotspot in the center was predicted to disappear, followed by the hotspot in the northwest.

Note the difference between Figures 2 and 3 with respect to counties with relative risk probability larger than one. Taking Week 20 as an example, Figure 2 indicates that, for example, the county in the center has relative risk probability larger than one (colored red) but Figure 3 shows that it is not a hotspot. The explanation is that the exceedance probability is smaller than 0.95 and thus does not qualify as a hotspot.

Figure 2 and in particular Figure 3 show that COVID‐19 in West Java is a local phenomenon concentrated in a limited number of counties ranging from 5 to 7 among 27 counties. The affected northwest part borders Jakarta Province, the country's outbreak epicenter. Intense commuting (work, shopping, entertainment, etc.) occurs between the northwestern counties and Jakarta Province. The hotspot in central West Java is the capital of the province, Bandung city. It has a high population density and a high commuting frequency with Jakarta, the nation's capital (see Tables B6 and B7 for detailed estimates and predictions). In the remainder of the province, the relative risk was low (less than one) and no hotspots were discovered. Note that the intensive commuting between Jakarta and the northwest and central West Java counties was hypothesized but not estimated because Jakarta does not belong to West Java Province.

The concentration of COVID‐19 in the northwest and center necessitates local measures, that is, inter‐county lockdowns, in particular restrictions for commuting to Jakarta as well as limitations for interactions of the hotspots with the other, not (yet) affected West Java counties. The persistence of the hotspots necessitates intra‐county lockdowns, such as closing public facilities, offices, and public transportation, limiting religious gatherings, social, and cultural activities, mandating wearing of face masks, and imposing social distancing measures. However, a province‐wide intra‐county lockdown is not required.

**Figure 4:**
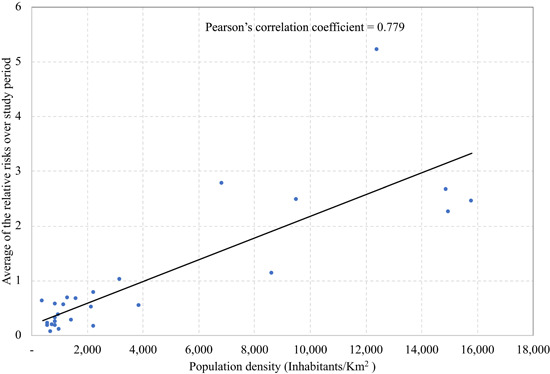
Scatterplot of population density versus the average of the estimated relative risk over the study period March 06–July 23, 2020 [Color figure can be viewed at wileyonlinelibrary.com]

Based on Figures 2 and 3, we noticed that the high‐risk areas are dominated by urban areas (cities) with high population density. To further examine the relationship we calculated the Pearson correlation between the population by land area in square kilometers and the average predicted relative risk over the study period. The result is presented in Figure 4. The Pearson correlation coefficient of 0.78 supports the hypothesis of a close relationship. This finding is in line with the growing evidence elsewhere that COVID‐19 infection is more likely in urban areas, which are densely populated, than in rural areas, which are less populated (Bhadra et al., 2020; Kadi & Khelfao, 2020; Saadat et al., 2020).

Regarding mobility and relative risk we refer to Table 1, where we observed a strong structured temporal effect indicating that the relative risk is an intra‐county issue in the first place. Regarding the structured spatial effect, we found a Leroux conditional autoregressive distribution with first‐order queen contiguity matrix and spatial autoregressive parameter $\hat{\rho }$ = 0.347, indicating that there is low spatial spillover among neighboring regions with a maximum distance of 42 km. The low spatial spillover effect is likely to be related to the fact that the city of Jakarta—the epicenter of the COVID‐19 disease in Indonesia—was not included in the analysis whereas there is intensive commuting between Jakarta and the provincial hotspots in the north‐east and the provincial capital Bandung in the center. Other reasons for the low spatial spillover effect are the large‐scale social restrictions (PSBB) that the provincial government of West Java implemented from April 22 to July 2, 2020 (Pangestika, 2020a, 2020b). PSBB allows the county governments to restrict the mobility of people and goods to and from their locations (Andriani, 2020).

## 4 DISCUSSION AND CONCLUSIONS

An early warning system for the control and management of the COVID‐19 pandemic requires mapping of the spatiotemporal dynamics of the disease and forecasting of the locations and times of future outbreaks. Herein, we propose the relative risk indicator as the key concept of such a warning system as it accounts for differences in the population at risk, such as the population size, which varies across space and time. Relative risk is based on the assumption that the number of incidences follows a Poisson distribution (or the Negative Binomial (NB) distribution or the zero‐inflated variants of the Poisson or NB distribution for the case of overdispersion). We observed that registration and analysis by age is a prerequisite to ensure a good understanding of the pandemic. The methodology presented in this paper can be straightforwardly adapted for characteristics of the population at risk, such as the age or sex structure.

We modeled the relative risk as a spatiotemporal pure model of the structured and unstructured spatial and temporal random effects and their interactions to capture unknown or unobserved risk factors. The model is parsimonious because it only requires the number of cases and the population at risk across space and time. Additionally, the pure model allows adequate mapping and forecasting of future outbreaks by location (county) and time (week).

A frequently occurring problem at fine spatiotemporal scales is that the estimators of the parameters have large standard errors because of data sparseness or spatiotemporal heterogeneity. The problem can be solved by smoothing, that is, by borrowing strength across spatiotemporal units such that the observations for a specific spatiotemporal unit are weighted toward the observations of neighboring units.

We demonstrated that the pure model can be conveniently estimated using Bayesian approaches, thereby allowing Leroux conditional spatial autoregressive based on first‐order queen contiguity, and random walk temporal priors to capture spatiotemporal dependence and heterogeneity. In addition, the Bayesian approach enables the relative risk uncertainty to be quantified via the posterior distributions, particularly the posterior exceedance probability (Adin et al., 2019) which can be used to identify hotspots, that is, isolated areas where the relative risk is unusually high. Hotspots are candidates for policy intervention.

The application to the West Java counties for the period March 6–July 9, 2020 demonstrated that the spatiotemporal distribution of COVID‐19 was primarily temporal, while there was relatively little spatial interaction between the West Java counties. The persistence of the hotspots over time indicated the necessity for intra‐county lockdowns. The pure model can be straightforwardly used to identify hotspots within a hotspot, such as districts or residential blocks. Local lockdowns or the imposition of mobility restrictions are effective policy instruments to combat the spread of the disease within a county.

The relatively low impact of the spatial component was probably due to (i) the fact that the city of Jakarta—the epicenter of the COVID‐19 disease in Indonesia—does not belong to the province of West Java, whereas there is intensive commuting between Jakarta and the provincial hotspots in the bordering counties in north‐east West Java and the provincial capital Bandung in the center and (ii) large‐scale social restrictions (PSBB) that West Java implemented from April 22 to July 2, 2020.

Taking into consideration the distance that the disease spread from Jakarta to the provincial hotspots, restrictions on ingoing and outgoing commute to Jakarta were needed. In a similar vein, restrictions on mobility between the West Java hotspots and the other counties in the province including low‐risk counties were necessary. Further analysis of the relative risk by county revealed that there is a close relationship with population density in line with the hypothesis that COVID‐19 infection is transmitted via interaction of noncontaminated people with infected people or via contact with contaminated objects and surfaces.

There are several limitations to the present study related to the data quality. First, this study is based on COVID‐19 cases reported by county. Due to different monitoring standards, this may have led to differences in incidence recording. Uniform data collection using international standards, multidisciplinary research, and combining statistical estimates with the outcomes of laboratory and field experiments are required for future research. Second, although the number of tests in West Java over the study period (March 06–July 09, 2020) was approximately constant at around 25,000 tests per week, it is important for future research to take changes in testing intensity into account. Third, improved monitoring could be achieved using longer time series data thus accommodating the different stages of the evolution of the pandemic (Dobricic et al., 2020). This kind of information presents clues to the effects of behavioral changes such as social distancing, working from home, closing schools, closing public facilities, offices, and public transportation, limiting religious gatherings, social events, and cultural activities, and wearing face masks. Finally, the analysis was restricted to the Province of West Java and excluded the heavy regular travel to Jakarta, the national epicenter of COVID‐19. An adequate early warning system requires data collection in functional regions and, if necessary, policy coordination among administrative regions (Yang, 2017).

In spite of the above‐mentioned shortcomings, the results of this study can still contribute to pandemic‐related policymaking at the county level because the inaccurate reporting of confirmed cases applies to each county. Additionally, predictions were based on the notion of relative risk, which is a more accurate measure to identify high‐risk counties than the number of confirmed cases, especially in the case of rare or sparse data (Tango, 2010; Tenny & Hoffman, 2020). In addition, supplementary analysis such as on the theoretically plausible and elsewhere confirmed correlation between population density and the location of the hotspots gives further support to the findings.

In summary, the hierarchical Bayesian pure spatiotemporal model is an easy to apply, widely applicable model for the accurate forecasting of short‐term spatiotemporal COVID‐19 relative risk. Additionally, it can be used to generate and test hypotheses regarding control, such as environmental or socioeconomic conditions.

## Data Availability

The data that support the findings of this study are available from the corresponding author upon reasonable request.

## APPENDIX A

### A1. Age‐structured effects

If age‐structured count data is available, the methodology presented in this paper can be straightforwardly adapted to include the age‐structured effects as follows. Assume that the region under study is divided into n counties and T periods with K age groups. Let $N_{\mathrm{itk}}$ denote the population at risk in county i at time t, and age group *k* and $y_{\mathrm{itk}}$ the number of confirmed COVID‐19 cases. Accordingly, $y_{\mathrm{itk}}$ follows a Poisson distribution with the mean and variance equal to $\lambda_{\mathrm{itk}}=E_{\mathrm{itk}}\theta_{i\mathrm{tk}}$, as follows:

$$y_{\mathrm{itk}}|\lambda_{\mathrm{itk}}\sim \text{Poisson}(E_{\mathrm{itk}}\theta_{\mathrm{itk}})$$

with $E_{\mathrm{itk}}$ and $\theta_{\mathrm{itk}}$ the expected number of cases and the relative risk in area i at time t for age group k, respectively. The expected number of cases $E_{\mathrm{itk}}$ is defined as (Wakefield, 2007):

$$E_{\mathrm{itk}}=N_{\mathrm{itk}}\times p_{k}$$

with $p_{k}$ the probability of COVID‐19 for age group k defined as

$$p_{k}=\frac{(\overset{n}{\underset{i=1}{\sum }}\overset{T}{\underset{t=1}{\sum }}y_{\mathrm{itk}})/\mathrm{nT}}{(\overset{n}{\underset{i=1}{\sum }}\overset{T}{\underset{t=1}{\sum }}N_{\mathrm{itk}})/\mathrm{nT}}=\frac{{\bar{y}}_{k}}{{\bar{N}}_{k}}.$$

To analyze the spatiotemporal distribution of COVID‐19 with age‐structured effects, Model (6) can be extended as follows (Lopez‐Quılez & Munoz, 2009):

(A1.1)
$$\eta_{\mathrm{itk}}=\alpha +\beta_{k}+\omega_{i}+\upsilon_{i}+\phi_{t}+\varphi_{t}+\partial_{\mathrm{ik}}+g_{\mathrm{tk}}+\delta_{\mathrm{it}}+\zeta_{\mathrm{ijk}}.$$

Here, $\eta_{\mathrm{itk}}=\log(\theta_{\mathrm{itk}})$. The additional components $\beta_{k}$, $\partial_{\mathrm{ik}},$ $g_{\mathrm{tk}}$, and $\zeta_{\mathrm{ijk}}$ denote the age‐structured effect for age group k, age–space interaction, age–time interaction, and age–space–time interaction, respectively. The last components $\zeta_{\mathrm{ijk}}$ is rarely used because it greatly increases the complexity of the model (Lopez‐Quılez & Munoz, 2009). The age‐structured effects can be modeled through a fixed effects or random effects model. Sun et al. (2000) and Xia and Carlin (1998) incorporated the age‐structured effects as fixed‐effects covariates through dummy variables. On the other hand, Knorr‐Held and Besag (1998) and Goicoa et al. (2016) treated the age‐structured effects as random effects, and applied the pure spatiotemporal model (Schmid & Held, 2004). According to Adin et al. (2019) and Jaya and Folmer (2020b), the random effects model is more appropriate to model age‐specific effects due to the identifiability problems of the fixed effects model caused by the large number of age‐group variables. This also applies to other group‐specific effects. In a Bayesian setting, the random effects component of the age‐structured effects $\beta_{k}$ can be modeled using random walk or exchangeable priors (Goicoa et al., 2016; Knorr‐Held & Besag, 1998). (Note that the age‐groups are indexed with an integer so that they can be directly modeled using random walk or exchangeable priors). A random walk prior of order one (RW1) $\beta_{k}$ reads:

$$\beta_{k+1}-\beta_{k}|\sigma_{\beta }^{2}\sim N\left(0,\sigma_{\beta }^{2}\right),\forall i,\mathrm{tk}=1,..,K$$

of order two (RW2) as

$$\beta_{k}-2\beta_{k+1}+\beta_{k+2}|\sigma_{\beta }^{2}\sim N\left(0,\sigma_{\beta }^{2},)\right),\;\forall i,t\;k=1,\ldots ,K.$$

An exchangeable prior reads as follows:

$$\beta_{k}|\sigma_{\beta }^{2}\sim N\left(0,\sigma_{\beta }^{2},)\right),\;\forall i,t\;k=1,\ldots ,K.$$

The interaction components $\partial_{\mathrm{ik}}\;\text{and}\;g_{\mathrm{tk}}$ may take four possible types, as introduced by Knorr‐Held (2000) (see Goicoa et al., 2016 for details). Model (A1.1) is estimated in a similar way as Model (6).

### A2. Integrated nested Laplace approximation (INLA)

Consider the pure Model (6). The joint posterior distribution of Ω and ψ given y, is (Rue et al., 2009):

(A2.1)
$$p\left((,\Omega ,\psi |y,)\right)=\frac{p\left((,y|\Omega ,\psi ,)\right)p\left((,\Omega |\psi ,)\right)p\left((,\psi ,)\right)}{p\left((,y|\psi ,)\right)}\propto p\left((,y|\Omega ,\psi ,)\right)p\left((,\Omega |\psi ,)\right)p\left((,\psi ,)\right),$$

where p(y|Ω,ψ) is the likelihood function of the number of cases, $y=(y_{11},\text{…},y_{nT})\prime ,i=1,\text{…},n$, and t=1,…,T which is based on the Poisson or Negative Binomial distribution, p(Ω|ψ) is the prior distribution of Ω given hyperparameter vector ψ, p(ψ) the joint hyperprior distribution of the hyperparameters ψ and p(y|ψ)is the marginal likelihood of the model which is a normalizing constant and usually ignored because of computational costs. The number of cases **y** is assumed independent given the parameters Ω and hyperparameter ψ. The likelihood function is then:

(A2.2)
$$p\left((,\mathrm{y|}\Omega ,\psi ,)\right)=\overset{n}{\underset{l=1}{\prod }}\overset{T}{\underset{t=1}{\prod }}p\left((,y_{\mathrm{it}}|\Omega_{\mathrm{it}},\psi ,)\right),$$

where each data point $y_{\mathrm{it}}$ is connected to only one element $\Omega_{\mathrm{it}}=(\alpha ,\omega_{i},\rho ,v_{i},\phi_{t},\varphi_{t},\delta_{\mathrm{it}})\prime$. p(Ω|ψ) is assumed to be the normal distribution. Hence:

(A2.3)
$$p\left((,\Omega |\psi ,)\right)\propto {\left|Q\left((,\psi ,)\right)\right|}^{\frac{1}{2}}\text{exp}\left\{-\frac{1}{2}\Omega^{\prime }Q\left((,\psi ,)\right)\Omega \right\},$$

where Q(ψ) is the precision matrix of ψ, that is, the inverse of the covariance matrix 𝚺(ψ). The elements of the hyperprior p(ψ) are assumed independent, and thus, they are regarded as the products of several univariate hyperpriors.

Inference in INLA is based on the marginal posterior distributions of the hyperparameters $\psi =\left((,\sigma_{\omega }^{2},\sigma_{\upsilon }^{2},\sigma_{\phi }^{2},\sigma_{\varphi }^{2},\sigma_{\delta }^{2},)\right)\prime$ and the main parameters of interest $\Omega =(\alpha ,\omega^{\prime },\rho ,v\prime ,\phi \prime ,\varphi \prime ,\delta \prime )\prime$. The aforenoted distributions of the former are needed to obtain the latter. The marginal posterior distribution of each hyperparameter *h*, where *h* = 1, …, 5, is

(A2.4)
$$p\left((,\psi_{h}|y,)\right)=\int p\left((,\psi |y,)\right)d\psi_{-h},$$

where $\psi_{-h}$ denotes all elements in ψ except the hth element.

To derive the marginal posterior distributions of the main parameter of interest, we first derive their joint posterior distribution at location i and time t, $\Omega_{\mathrm{it}}$:

(A2.5)
$$p\left((,\Omega_{\text{it}}|y,)\right)=\int p\left((,\Omega_{\text{it}},\psi \mathrm{|y},)\right)d\psi =\int p\left((,\Omega_{\text{it}}|\psi ,y,)\right)p\left((,\psi |y,)\right)d\psi .$$

The joint posterior distribution p(ψ|y) in Equation (A2.5) can be approximated as (Rue et al., 2009):

(A2.6)
$$\tilde{p}\left((,\psi |y,)\right)\approx {\left(\frac{p\left((,\Omega ,\psi |y,)\right)}{\tilde{p}\left((,\Omega |\psi ,y,)\right)}\right|}_{\Omega =\Omega^{⁎}\left((,\psi ,)\right)},$$

where $\tilde{p}(\Omega |\psi ,y)$ is the Gaussian approximation to the full conditional distribution of Ω, obtained via Laplace approximation and $\Omega^{⁎}(\psi )=\text{arg}\text{ma}x_{\Omega }\tilde{p}(\Omega |\psi ,y)$is the posterior mode of the full conditional distribution $\tilde{p}(\Omega |\psi ,y)$ for given ψ. The posterior mode, referred to as the maximum a‐posteriori (MAP) estimator (Gelman et al., 2013), is obtained by taking $\tilde{p}(\Omega |\psi ,y)$ proportional to the likelihood function p(Ω|ψ,y) weighted by the Gaussian approximation to the prior distribution $\tilde{p}(\Omega |\psi )$ and the hyperprior distribution p(ψ) using Bayes' theorem, as follows:

(A2.7)
$$\Omega^{⁎}\left((,\psi ,)\right)=\text{arg}\;\text{ma}x_{\Omega }\tilde{p}\left((,\Omega |\psi ,y,)\right)=\text{arg}\;\text{ma}x_{\Omega }\frac{p\left((,y|\Omega ,\psi ,)\right)\tilde{p}\left((,\Omega |\psi ,)\right)p\left((,\psi ,)\right)}{p\left((,y|\psi ,)\right)}\propto \text{arg}\;\text{ma}x_{\Omega }p\left((,y|\Omega ,\psi ,)\right)\tilde{p}\left((,\Omega |\psi ,)\right)p\left((,\psi ,)\right),$$

where p(y|ψ) can be ignored because p(y|ψ) does not depend on Ω. MAP estimates of the posterior mode $\Omega^{⁎}(\psi )$ can be computed via numerical optimization, such as Newton's method (Rue et al., 2009).

The joint posterior of the model parameters $p(\Omega_{\text{it}}|\psi ,y)$ in Equation (A2.5) is approximated as

(A2.8)
$$\tilde{p}\left((,\Omega_{\mathrm{it}}|\psi ,y,)\right)\approx {\left(\frac{p\left((,\left((,\Omega_{\mathrm{it}},\Omega_{-\mathrm{it}},)\right),\psi \mathrm{|y},)\right)}{\tilde{p}\left((,\Omega_{-\mathrm{it}}|\Omega_{\mathrm{it}},\psi ,y,)\right)}\right|}_{\Omega_{-\mathrm{it}}=\Omega_{-\mathrm{it}}^{⁎}\left((,\psi ,)\right)},$$

where $\Omega_{-\mathrm{it}}$ denotes all the elements in Ω except the it‐th element, $\tilde{p}(\Omega_{-\mathrm{it}}|\Omega_{\mathrm{it}},\psi ,y)$ is the Laplace Gaussian approximation to $p(\Omega_{-\mathrm{it}}|\Omega_{\mathrm{it}},\psi ,y)$ and $\Omega_{-\mathrm{it}}=\Omega_{-\mathrm{it}}^{⁎}\left((,\psi ,)\right)$ is its mode. Given $\tilde{p}(\Omega_{\mathrm{it}}|\psi ,y)$ and $\tilde{p}(\psi |y)$ the marginal posterior distribution of $p(\Omega_{\mathrm{it}}|y)$ can be approximated as:

(A2.9)
$$\tilde{p}\left((,\Omega_{\mathrm{it}}\mathrm{|y},)\right)\approx \int \tilde{p}\left((,\Omega_{\mathrm{it}}|\psi ,y,)\right)\tilde{p}\left((,\psi |y,)\right)d\psi ,$$

where the integral Equation (A2.9) is solved numerically through a finite weighted sum:

(A2.10)
$$\tilde{p}\left((,\Omega_{\mathrm{it}}\mathrm{|y},)\right)\approx \overset{J}{\underset{j}{\sum }}\tilde{p}\left((,\Omega_{\mathrm{it}}|\psi^{\left(j\right)},y,)\right)\tilde{p}\left((,\psi^{\left(j\right)}|y,)\right){∆}_{j},$$

where {$\psi^{\left(j\right)}$} is a set of values of ψ associated with the integration weights ${∆}_{j}$; further, *J* denotes the number of evaluation points (Rue et al., 2009). INLA encompasses three alternative approaches to obtain the integration points. The first builds a regular two‐dimensional spatial grid on ψ around the posterior mode of $\tilde{p}(\psi |y)$. This yields an accurate approximation but the number of grid points increases exponentially with the size of ψ. The second approach uses a central composite design with fewer points around the posterior mode of $\tilde{p}(\psi |y)$; however, it is less accurate than the first one. The last approach, the empirical Bayes approach, ignores the variability of the hyperparameter and just uses the posterior mode of $\tilde{p}(\psi |y)$ (Rue et al., 2009).

The marginal posterior densities of the parameters of interest *k*, where *k =* 1,…,6, can be used to obtain approximate summary statistics, such as the posterior mean as follows. Consider the observed data $y=(y_{11},\ldots ,y_{\mathrm{nT}})\prime$ from the distribution $p(y_{\mathrm{it}}|\Omega_{\mathrm{it}},\psi ),$ with parameter $\Omega_{\mathrm{it}}$ and hyperparameter ψ and Gaussian approximation $\tilde{p}(\Omega_{\mathrm{it}})$to the prior distribution $p(\Omega_{\mathrm{it}})$,. Here, the marginal posterior density of $\Omega_{\mathrm{kit}}$ given the observation **y** is $\tilde{p}\left((,\Omega_{\mathrm{kit}}|y,)\right)=\int \tilde{p}\left((,\Omega_{\mathrm{it}}|y,)\right)d\Omega_{-\mathrm{kit}}$, where $\Omega_{-\mathrm{kit}}$ denotes the $\Omega_{\mathrm{it}}$ without the kth element. The posterior mean of the kth parameter is

(A2.11)
$$E\left((,\Omega_{\mathrm{kit}}|y,)\right)=\int \Omega_{\mathrm{kit}}\tilde{p}\left((,\Omega_{\mathrm{kit}}|y,)\right)d\Omega_{\mathrm{kit}},\;k=1,\ldots ,6,\;i=1,\ldots ,n\;\text{and}\;t=1,\ldots ,T.$$

The integral Equation (A2.11) can be approximated using the Laplace approximation and Bayes' theorem to obtain:

(A2.12)
$$\int \Omega_{\mathrm{kit}}\tilde{p}\left((,\Omega_{\mathrm{kit}}|y,)\right)d\Omega_{\mathrm{kit}}=\int \Omega_{\mathrm{kit}}\frac{p\left((,\mathrm{y|}\Omega_{\mathrm{kit}},)\right)\tilde{p}\left((,\Omega_{\mathrm{kit}},)\right)}{p\left((,y,)\right)}d\Omega_{\mathrm{kit}}=\int \Omega_{\mathrm{kit}}\frac{p\left((,\mathrm{y|}\Omega_{\mathrm{kit}},)\right)\tilde{p}\left((,\Omega_{\mathrm{kit}},)\right)}{\int p\left((,\mathrm{y|}\Omega_{\mathrm{kit}},)\right)\tilde{p}\left((,\Omega_{\mathrm{kit}},)\right)d\Omega_{\mathrm{kit}}}d\Omega_{\mathrm{kit}}=\frac{\int \Omega_{\mathrm{kit}}\text{exp}\left((,\log\left((,p\left((,y|\Omega_{\mathrm{kit}},)\right)\tilde{p}(\Omega_{\mathrm{kit}}),)\right),)\right)d\Omega_{\mathrm{kit}}}{\int \text{exp}\left((,\log\left((,p\left((,y|\Omega_{\mathrm{kit}},)\right)\tilde{p}(\Omega_{\mathrm{kit}}),)\right),)\right)d\Omega_{\mathrm{kit}}}.$$

Let $h(\Omega_{\mathrm{kit}})=\log(p(y|\Omega_{\mathrm{kit}})\tilde{p}(\Omega_{\mathrm{kit}})).$ Then Equation (A2.12) becomes

(A2.13)
$$\int \Omega_{\mathrm{kit}}\tilde{p}\left((,\Omega_{\mathrm{kit}}|y,)\right)d\Omega_{\mathrm{kit}}=\frac{\int \Omega_{\mathrm{kit}}\text{exp}\left((,h\left((,\Omega_{\mathrm{kit}},)\right),)\right)d\Omega_{\mathrm{kit}}}{\int \text{exp}\left((,h\left((,\Omega_{\mathrm{kit}},)\right),)\right)d\Omega_{\mathrm{kit}}}.$$

Equation (A2.13) can be solved using the Laplace approximation as follows. Because it is a monotonic transformation of a function proportional to the posterior density $\tilde{p}(\Omega_{\mathrm{kit}}|y)$, $h(\Omega_{\mathrm{kit}})$ achieves its maximum at the a‐posteriori mode ${\hat{\Omega }}_{\mathrm{kit}}=\text{arg}\;\text{ma}x_{\Omega_{\mathrm{kit}}}\tilde{p}(\Omega_{\mathrm{kit}}|y)$. Taking the Taylor series approximation of $h(\Omega_{\mathrm{kit}})$ around ${\hat{\Omega }}_{\mathrm{kit}}$ gives

(A2.14)
$$h\left((,\Omega_{\mathrm{kit}},)\right)\approx h\left((,{\hat{\Omega }}_{\mathrm{kit}},)\right)+h'\left((,{\hat{\Omega }}_{\mathrm{kit}},)\right)\left((,\Omega_{\mathrm{kit}}-{\hat{\Omega }}_{\mathrm{kit}},)\right)+\frac{1}{2}h"({\hat{\Omega }}_{\mathrm{kit}}){\left((,\Omega_{\mathrm{kit}}-{\hat{\Omega }}_{\mathrm{kit}},)\right)}^{2}.$$

Because ${\hat{\Omega }}_{\mathrm{kit}}$ is the posterior mode of $\tilde{p}(\Omega_{\mathrm{kit}}|y),h^{\prime }({\hat{\Omega }}_{\mathrm{kit}})=0$. Hence,

(A2.15)
$$h\left((,\Omega_{\mathrm{kit}},)\right)\approx h\left((,{\hat{\Omega }}_{\mathrm{kit}},)\right)+\frac{1}{2}h"({\hat{\Omega }}_{\mathrm{kit}}){\left((,\Omega_{\mathrm{kit}}-{\hat{\Omega }}_{\mathrm{kit}},)\right)}^{2},$$

and Equation (A2.13) can now be written as

(A2.16)
$$E\left((,\Omega_{\mathrm{kit}}|y,)\right)=\int \Omega_{\mathrm{kit}}\tilde{p}\left((,\Omega_{\mathrm{kit}}|y,)\right)d\Omega_{\mathrm{kit}}=\frac{\int \Omega_{\mathrm{kit}}\text{exp}\left((,h\left((,{\hat{\Omega }}_{\mathrm{kit}},)\right)+\frac{1}{2}h"({\hat{\Omega }}_{\mathrm{kit}}){\left((,\Omega_{\mathrm{kit}}-{\hat{\Omega }}_{\mathrm{kit}},)\right)}^{2},)\right)d\Omega_{\mathrm{kit}}}{\int \text{exp}\left((,h\left((,{\hat{\Omega }}_{\mathrm{kit}},)\right)+\frac{1}{2}h"({\hat{\Omega }}_{\mathrm{kit}}){\left((,\Omega_{\mathrm{kit}}-{\hat{\Omega }}_{\mathrm{kit}},)\right)}^{2},)\right)d\Omega_{\mathrm{kit}}}=\frac{\text{exp}\left((,h\left((,{\hat{\Omega }}_{\mathrm{kit}},)\right),)\right)\int \Omega_{\mathrm{kit}}\text{exp}\left((,\frac{1}{2}h"({\hat{\Omega }}_{\mathrm{kit}}){\left((,\Omega_{\mathrm{kit}}-{\hat{\Omega }}_{\mathrm{kit}},)\right)}^{2},)\right)d\Omega_{\mathrm{kit}}}{\text{exp}\left((,h\left((,{\hat{\Omega }}_{\mathrm{kit}},)\right),)\right)\int \text{exp}\left((,\frac{1}{2}h"({\hat{\Omega }}_{\mathrm{kit}}){\left((,\Omega_{\mathrm{kit}}-{\hat{\Omega }}_{\mathrm{kit}},)\right)}^{2},)\right)d\Omega_{\mathrm{kit}}}=\frac{\text{exp}\left((,h\left((,{\hat{\Omega }}_{\mathrm{kit}},)\right),)\right)\sqrt{\frac{2\pi }{-h"({\hat{\Omega }}_{\mathrm{kit}})}}\int \Omega_{\mathrm{kit}}{\left(\sqrt{\frac{2\pi }{-h"({\hat{\Omega }}_{\mathrm{kit}})}},)\right)}^{-1}\text{exp}\left(-\frac{{\left((,\Omega_{\mathrm{kit}}-{\hat{\Omega }}_{\mathrm{kit}},)\right)}^{2}}{2(-{h"({\hat{\Omega }}_{\mathrm{kit}})}^{-1})},)\right)d\Omega_{\mathrm{kit}}}{\text{exp}\left((,h\left((,{\hat{\Omega }}_{\mathrm{kit}},)\right),)\right)\sqrt{\frac{2\pi }{-h"({\hat{\Omega }}_{\mathrm{kit}})}}\int {\left(\sqrt{\frac{2\pi }{-h"({\hat{\Omega }}_{\mathrm{kit}})}},)\right)}^{-1}\text{exp}\left((,\frac{1}{2}h"({\hat{\Omega }}_{\mathrm{kit}}){\left((,\Omega_{\mathrm{kit}}-{\hat{\Omega }}_{\mathrm{kit}},)\right)}^{2},)\right)d\Omega_{\mathrm{kit}}}=\frac{\int \Omega_{\mathrm{kit}}\tilde{p}\left((,\Omega_{\mathrm{kit}}|{\hat{\Omega }}_{\mathrm{kit}},\sigma^{2},)\right)d\Omega_{\mathrm{kit}}}{\int \tilde{p}\left((,\Omega_{\mathrm{kit}}|{\hat{\Omega }}_{\mathrm{kit}},\sigma^{2},)\right)d\Omega_{\mathrm{kit}}},$$

where $\sigma^{2}=-{\mathrm{h"}({\hat{\Omega }}_{\mathrm{kit}})}^{-1}$ and $\int \tilde{p}\left((,\Omega_{\mathrm{kit}}|{\hat{\Omega }}_{\mathrm{kit}},\sigma^{2},)\right)d\Omega_{\mathrm{kit}}=1$ owing to the property that the integral of a density function over the entire space yields 1. Hence, Equation (A2.16) becomes:

(A2.17)
$$E\left((,\Omega_{\mathrm{kit}}|y,)\right)=\int \Omega_{\mathrm{kit}}\tilde{p}\left((,\Omega_{\mathrm{kit}}|{\hat{\Omega }}_{\mathrm{kit}},\sigma^{2},)\right)d\Omega_{\mathrm{kit}}.$$

Because the expectation of a Gaussian distribution is equal to the mode we have:

(A2.18)
$$E(\Omega_{\mathrm{kit}}|y)={\hat{\Omega }}_{\mathrm{kit}},\;k=1,\ldots 6,\;i=1,\ldots n\;\text{and}\;t=1,\ldots ,T.$$

The estimated posterior mean values defined by (A2.18) yield the posterior mean of the relative risk at location i and time *t* as follows: ${\hat{\theta }}_{\mathrm{it}}=\text{exp}(\hat{\alpha }+{\hat{\omega }}_{i}+{\hat{\upsilon }}_{i}+{\hat{\phi }}_{t}+{\hat{\gamma }}_{t}+{\hat{\delta }}_{\mathrm{it}})$. Note that posterior mean of the intercept is constant over space and time; the posterior means of the structured and unstructured temporal effects are constant over space; the posterior means of the structured and unstructured spatial effects are constant over time; and the posterior means of the interaction effects vary over space and time.

Forecasting in a Bayesian framework is out‐of‐bounds prediction based on the posterior predictive distribution $p({\hat{y}}_{\left(T+h\right)}\mathrm{|y})$ of the unobserved quantity, ${\hat{y}}_{\left(T+h\right)}$, given the observed data **y**, with h=1,2,… steps ahead from T. In particular (Jaya & Folmer, 2020a):

(A2.19)
$$p\left((,{\hat{y}}_{\left(T+h\right)}\mathrm{|y},)\right)=\int p\left((,{\hat{y}}_{\left(T+h\right)}|\psi ,y,)\right)p\left((,\psi \mathrm{|y},)\right)d\psi ,$$

where the marginal posterior distribution for a *h*‐step‐ahead forecast $p({\hat{y}}_{\left(T+h\right)}|\psi ,y)$ is obtained by integrating the posterior conditional distribution $p({\hat{y}}_{\left(T+h\right)},\Omega |\psi ,y)$ over Ω:

(A2.20)
$$p\left((,{\hat{y}}_{\left(T+h\right)}|\psi ,y,)\right)=\int p\left((,{\hat{y}}_{\left(T+h\right)},\Omega |\psi ,y,)\right)d\Omega =\int p\left((,{\hat{y}}_{\left(T+h\right)}|\Omega ,\psi ,)\right)p\left((,\Omega |\psi ,y,)\right)d\Omega .$$

The numerical method outlined in (A2.10) can be used to approximate the integral over hyperparameters ψ in (A2.19). The desired out‐of‐bounds prediction is entered into the INLA software as a not available (NA) observation. The prediction is generated when the model is fitted (X. Wang et al., 2018).

## APPENDIX B

**Table B1:** The county ids, name of the corresponding county, population at risk, and population density, West Java province, 2019

*ids*	County	Status	Population	Population density (1000 Inhabitants/km^2^)
*1*	Bogor regency	Rural	5,965,410	2.201
*2*	Sukabumi regency	Rural	2,466,272	0.595
*3*	Cianjur regency	Rural	2,263,072	0.589
*4*	Bandung regency	Rural	3,775,279	2.135
*5*	Garut regency	Rural	2,622,425	0.853
*6*	Tasikmalaya regency	Rural	1,754,128	0.688
*7*	Ciamis regency	Rural	1,195,176	0.845
*8*	Kuningan regency	Rural	1,080,804	0.973
*9*	Cirebon regency	Rural	2,192,903	2.227
*10*	Majalengka regency	Rural	1,205,034	1.001
*11*	Sumedang regency	Rural	1,152,400	0.759
*12*	Indramayu regency	Rural	1,728,469	0.847
*13*	Subang regency	Rural	1,595,825	0.843
*14*	Purwakarta regency	Rural	962,893	1.166
*15*	Karawang regency	Rural	2,353,915	1.425
*16*	Bekasi regency	Rural	3,763,886	3.073
*17*	Bandung Barat regency	Rural	1,699,896	1.302
*18*	Pangandaran regency	Rural	399,284	0.395
*19*	Bogor city	Urban	1,112,081	9.385
*20*	Sukabumi city	Urban	328,680	6.812
*21*	Bandung city	Urban	2,507,888	14.957
*22*	Cirebon city	Urban	319,312	8.547
*23*	Bekasi city	Urban	3,003,923	14.539
*24*	Depok city	Urban	2,406,826	12.017
*25*	Cimahi city	Urban	614,304	15.643
*26*	Tasikmalaya city	Urban	663,517	3.866
*27*	Banjar city	Urban	183,110	1.613

**Table B2:** Comparison of six variants of Model (6) with RW1 and RW2 trends for the Poisson, zero‐inflated Poisson (ZIP), negative binomial (NB), and zero‐inflated negative binomial (ZINB) distribution

Statistics	Model^a^	RW1				RW2			
		Poisson	NB	ZIP	ZINB	Poisson	NB	ZIP	ZINB
DIC	M1	3165.25	2113.08	3084.66	2114.25	3165.88	2114.18	3085.07	2113.68
	M2	3164.92	2114.08	3084.38	2113.96	3164.96	2113.59	3084.38	2114.47
	M3	1808.14	1874.24	2287.03	2068.51	1809.95	2021.70	2296.84	2067.23
	M4	1848.94	2107.34	2507.04	2112.52	1927.24	2113.41	2395.85	2123.19
	M5	1817.29	2072.79	2292.02	2056.10	1819.60	2081.29	2279.23	2065.48
	M6	1840.61	2085.79	2091.56	2084.74	9489.57	2094.84	2226.17	2107.00
WAIC	M1	3503.52	2122.45	3425.49	2121.80	3501.63	2120.48	3423.81	2121.38
	M2	3504.17	2122.16	3425.89	2123.26	3502.99	2120.97	3424.40	2121.68
	M3	1767.44	1845.75	2501.29	2073.47	1775.70	2002.93	2516.54	2063.58
	M4	1848.71	2112.74	2824.83	2117.81	2012.97	2117.91	2726.98	2121.89
	M5	1797.47	2075.42	2511.85	2049.95	1806.55	2079.71	2493.54	2047.17
	M6	1863.34	2099.94	2144.28	2097.55	8.52E+15	2112.77	2566.97	2112.62
MPL	M1	−1275.26	−769.47	−1265.60	−771.13	−1273.74	−769.65	−1263.52	−770.39
	M2	−1275.79	−770.35	−1266.11	−770.30	−1275.07	−769.54	−1265.55	−770.50
	M3	−1308.91	−773.71	−886.87	−866.87	−1317.97	−786.84	−1004.62	−1004.60
	M4	−1172.53	−775.98	−910.92	−883.53	−1419.71	−798.79	−1161.64	−806.03
	M5	−1430.00	−1039.95	−1462.47	−932.92	−1460.89	−1018.29	−1458.72	−917.79
	M6	−1345.46	−868.00	−1457.30	−801.97	−1719.76	−864.09	−1453.77	−855.51
MAE	M1	6.20	6.10	24.89	23.03	6.52	6.49	21.98	18.85
	M2	6.09	6.02	25.88	24.03	6.65	6.64	17.45	15.92
	M3	6.02	6.08	24.90	23.72	6.74	6.72	14.48	14.54
	M4	10.77	5.32	97.55	59.02	978.13	10.24	245.83	168.08
	M5	5.52	5.90	63.83	23.14	6.22	6.47	63.59	16.43
	M6	6.81	5.54	63.53	27.53	9.83E+18	5.97	63.83	41.06
RMSE	M1	15.20	15.15	61.03	55.22	15.85	15.89	57.69	47.72
	M2	15.01	14.99	62.42	56.52	16.06	16.16	42.89	38.66
	M3	15.05	15.15	55.56	55.98	16.25	16.23	33.81	34.16
	M4	21.17	11.20	247.83	131.00	4288.08	22.01	906.54	410.64
	M5	13.13	13.84	114.25	54.18	15.31	15.73	114.25	39.22
	M6	13.58	13.54	114.62	68.50	7.45E+19	15.01	115.50	100.43
*r*	M1	0.57	0.60	0.52	0.53	0.49	0.52	0.45	0.46
	M2	0.60	0.61	0.53	0.54	0.56	0.56	0.50	0.49
	M3	0.62	0.62	0.54	0.55	0.58	0.57	0.52	0.52
	M4	0.62	0.74	0.42	0.61	0.01	0.65	0.15	0.47
	M5	0.42	0.39	0.37	0.31	0.60	0.63	0.37	0.51
	M6	0.76	0.68	0.37	0.59	0.01	0.58	0.37	0.63

a M1: Structured spatial + structured temporal random effects. M2: Structured spatial + structured temporal + unstructured spatial + unstructured temporal random effects. M3: M2 + unstructured spatial × unstructured temporal interaction. M4: M2 + unstructured spatial × structured temporal interaction. M5: M2 + structured spatial × unstructured temporal interaction. M6: M2 + structured spatial × structured temporal interaction.

**Table B3:** Deviance index of the RW1 Poisson models and posterior mean, standard error (*SE*), and credible interval (CI) of the overdispersion parametersof the RW1 NB models

			NB Overdispersion parameter estimate		
No	Model	Deviance index^a^	Mean	SE	95% CI
(M1)	Structured spatial + structured temporal effects	3.256	1.478	0.1881	(1.106; 1.837)
(M2)	Structured spatial + structured temporal + unstructured spatial + unstructured temporal effects	3.261	1.475	0.1685	(1.165; 1.826)
(M3)	M2 + interaction type I	1.776	1582.794	3.20E+04	(21.476; 9445.170)
(M4)	M2 + interaction type II	3.145	1.644	0.2323	(1.635; 2.130)
(M5)	M2 + interaction type III	2.467	2.382	0.569	(1.417; 3.643)
(M6)	M2 + interaction type IV	2.890	2.093	0.2887	(2.092; 2.668)

^a^ The deviance index is defined as: DevianceIndex=D/(df−1), where *D* is the deviance statistic $D=2\left\{\overset{n}{\underset{i=1}{\sum }}\overset{T}{\underset{t=1}{\sum }}y_{\mathrm{it}}\log(y_{\mathrm{it}}/{\hat{\lambda }}_{\mathrm{it}})-\left(y_{\mathrm{it}}-{\hat{\lambda }}_{\mathrm{it}}\right)\right\}$, with ${\hat{\lambda }}_{\mathrm{it}}={\hat{\theta }}_{\mathrm{it}}E_{\mathrm{it}}$ and df the degrees of freedom defined as *df* = nT − pD with pD denoting the effective number of parameters. A deviance index greater than 1 suggests overdispersion (Mohebbi et al., 2014).

### B.1 B1. Comparison CAR, SAR, and LEROUX

We considered the following precision matrices $Q_{\omega }$ of the spatially structured random effect ω in (7), with joint prior distribution:

$$p\left((,\omega ,|,\sigma_{\omega }^{2},)\right)\propto {\left((,\sigma_{\omega }^{2},)\right)}^{-\frac{n-1}{2}}\exp\left(-\frac{1}{2}\omega^{'}Q_{\omega }\omega ,)\right).$$

(a) The Conditional autoregressive (CAR) distribution with $Q_{\omega }$ defined as
  $$Q_{\omega }=(1-\rho W),$$
  where $W=(w_{\mathrm{ij}})$ denotes the n×n spatial weights matrix with n the number of spatial units and ρ the spatial autoregressive parameter.
(b) The intrinsic conditional autoregressive (iCAR) distribution with $Q_{\omega }$ defined as
  $$Q_{\omega }=\mathrm{diag}(n_{i})-W,$$
  with $n_{i}$ is the number of neighbors of area i
(c) The Leroux CAR distribution with $Q_{\omega }$ defined as
  $$Q_{\omega }=[(1-\rho )I_{n}+\rho M],$$
  where $I_{n}$ is the n×n identity matrix and $M=\mathrm{diag}(n_{i})-W$
(d) The simultaneous autoregressive/spatial error model (SAR) with $Q_{\omega }$ defined as

$$Q_{\omega }=[\left(1-\rho W)'(1-\rho W\right)].$$

We estimated Model M4 using the Leroux CAR (LCAR), CAR and SAR priors and found that that in general, the former performed best with the smallest DIC and WAIC. See Table B4.

**Table B4:** Model comparison based on the LCAR, CAR, and SAR prior

Prior	DIC	WAIC
LCAR	2107.343	2112.742
CAR	2113.491	2117.178
SAR	2112.960	2116.580

### B.2 B2. Comparison spatial weights matrices

We explored the following alternative spatial weights matrices based on geographical distance ${(d}_{\mathrm{ij}})$ between areas i and j measured as the Euclidean distance between their respective centroids.

(a) Fixed distance
  $$w_{\mathrm{ij}}=\left\{\{,\begin{array}{cc} 1 & 0<d_{\mathrm{ij}}\leq d\\ 0 & \mathrm{otherwise}. \end{array}\right)$$
(b) Inverse distance power function
  $$w_{\mathrm{ij}}={\left(\frac{1}{d_{\mathrm{ij}}}\right)}^{p}$$
  for positive integer p, with p taken to be 1 or 2.
(c) Exponential distance
  $$w_{\mathrm{ij}}=\text{exp}(-\lambda d_{\mathrm{ij}})$$
  with λ taken to be 1 (Fahrmeir & Kneib, 2011)
(d) The combination of inverse distance and fixed distance
  $$w_{\mathrm{ij}}=\left\{\{,\begin{array}{cc} {\left(\frac{1}{d_{\mathrm{ij}}},)\right)}^{p} & 0<d_{\mathrm{ij}}\leq d\\ 0 & \text{otherwise}. \end{array}\right)$$
(e) The combination of inverse distance and first‐order queen contiguity
  $$w_{\mathrm{ij}}=\left\{\{,\begin{array}{cl} {\left(\frac{1}{d_{\mathrm{ij}}},)\right)}^{p} & \text{if}\;\text{areas}\;i\;\text{and}\;j\;\text{share}\;a\;\text{common}\;\text{border}\;\text{or}\;\text{vertix}\\ 0 & \text{otherwise}. \end{array}\right)$$
(f) The combination of exponential distance and fixed distance
  $$w_{\mathrm{ij}}=\left\{\{,\begin{array}{cl} \text{exp}\left((,-\lambda d_{ij},)\right) & 0<d_{ij}\leq d\\ 0 & \text{otherwise}. \end{array}\right)$$
(g) The combination of exponential distance and first‐order queen contiguity

$$w_{\mathrm{ij}}=\left\{\{,\begin{array}{cl} \text{exp}\left((,-\lambda d_{\mathrm{ij}},)\right) & \text{if}\;\text{areas}\;i\;\text{and}\;j\;\text{share}\;a\;\text{common}\;\text{border}\;\text{or}\;\text{vertix}\\ 0 & \text{otherwise}. \end{array},)\right)$$

We defined three different thresholds d = {42, 75, 150} roughly indicating daily, weekly, and monthly commuting time by car or bus, respectively. The minimum threshold *d *= 42 corresponded to the largest first‐order nearest neighbor distance. We evaluated the spatial weights matrices **W** for Model M4 using the following model selection criteria: the deviance information criterion (DIC), the Watanabe–Akaike information criterion (WAIC), the marginal predictive likelihood (MPL), mean absolute error (MAE), the root mean square error (RMSE), the correlation between observed and predicted values (*r*), and the spatiotemporal Moran I statistic (MoranST) for residual spatiotemporal autocorrelation (see Jaya and Folmer (2020b) for details on the MoranST). We ran the 15 models in INLA, and report the results in Table B5.

**Table B5:** Comparison of Model M4 based on queen contiguity, fixed distance, inverse distance, exponential distance with thresholds *d *= 42 km, *d *= 75 km, and *d *= 150 km, and the combination queen contiguity and distance, respectively^a^

Spatial weights matrix		$\hat{\rho }$	DIC	WAIC	MPL	MAE	RMSE	*r*	Moran ST
First‐order queen contiguity	W1	0.347	2107.343	2112.742	−775.984	5.320	11.198	0.743	–0.076
Fixed distance with threshold *d *= 42	W2	0.317	2110.283	2115.611	–848.426	5.115	12.212	0.746	–0.116
Fixed distance with threshold *d *= 75	W3	0.394	2108.586	2115.944	–782.634	5.778	11.240	0.746	–0.032
Fixed distance with threshold *d *= 150	W4	0.454	2109.049	2114.655	–776.795	5.153	11.358	0.735	–0.033
Inverse distance with threshold *d *= 42 (*p *= 1)	W5	0.238	2112.522	2116.688	–771.542	5.045	12.466	0.712	–0.158
Inverse distance with threshold *d *= 75 (*p *= 1)	W6	0.269	2109.582	2114.513	–781.957	5.273	11.275	0.738	–0.169
Inverse distance with threshold *d *= 150 (*p *= 1)	W7	0.267	2108.480	2112.837	–784.095	5.492	11.219	0.742	–0.172
Inverse distance with threshold *d *= 42 (*p *= 2)	W8	0.137	2108.774	2115.469	–853.486	5.115	12.203	0.747	–0.171
Inverse distance with threshold *d *= 75 (*p *= 2)	W9	0.235	2113.773	2117.312	–789.928	5.542	11.183	0.744	–0.154
Inverse distance with threshold *d *= 150 (*p *= 2)	W10	0.206	2110.292	2114.494	–782.041	5.671	11.218	0.745	–0.174
Exponential with threshold *d *= 42	W11	0.203	2109.800	2115.360	–806.674	5.114	12.875	0.720	–0.183
Exponential with threshold *d *= 75	W12	0.222	2110.270	2114.590	–770.899	5.588	11.249	0.743	–0.188
Exponential with threshold *d *= 150	W13	0.195	2108.297	2115.253	–777.486	5.156	11.370	0.734	–0.187
Inverse distance with first‐order queen contiguity	W14	0.433	2111.200	2115.930	–782.834	5.433	11.200	0.742	–0.099
Exponential with first‐order contiguity	W15	0.215	2113.794	2117.383	–775.322	5.367	11.276	0.738	–0.165

^a^ The estimated spatial autocorrelation coefficient $\hat{\rho }$ for LCAR ranges from 0.137 to 0.454. The model selection criteria show that there is no uniformly best model. W1–W4 perform approximately equally. Hence, we selected W1 for further analysis.

**Table B6:** Estimated (Weeks 1–18) and predicted (Weeks 19–20) relative risk by county^a^

		Week																			
*ids*	County	1	2	3	4	5	6	7	8	9	10	11	12	13	14	15	16	17	18	19	20
*1*	Bogor regency	0.09	0.14	0.18	0.54	0.64	0.76	0.77	1.00	1.25	0.94	1.49	0.95	0.59	0.87	0.93	0.94	1.17	1.16	0.74	0.39
*2*	Sukabumi regency	0.02	0.04	0.05	0.15	0.18	0.22	0.23	0.32	0.39	0.27	0.40	0.25	0.15	0.22	0.25	0.26	0.33	0.33	0.21	0.11
*3*	Cianjur regency	0.02	0.03	0.03	0.10	0.12	0.15	0.16	0.22	0.29	0.22	0.33	0.20	0.12	0.18	0.20	0.21	0.28	0.28	0.18	0.10
*4*	Bandung regency	0.06	0.11	0.13	0.38	0.46	0.55	0.55	0.71	0.85	0.59	0.86	0.54	0.33	0.49	0.59	0.63	0.82	0.85	0.54	0.29
*5*	Garut regency	0.02	0.03	0.04	0.10	0.12	0.15	0.15	0.21	0.27	0.21	0.34	0.23	0.15	0.20	0.21	0.21	0.26	0.25	0.16	0.09
*6*	Tasikmalaya regency	0.01	0.01	0.01	0.04	0.04	0.05	0.05	0.07	0.09	0.06	0.10	0.06	0.04	0.06	0.07	0.08	0.10	0.10	0.06	0.03
*7*	Ciamis regency	0.02	0.04	0.05	0.15	0.17	0.21	0.21	0.29	0.36	0.27	0.41	0.26	0.17	0.26	0.31	0.34	0.46	0.48	0.30	0.16
*8*	Kuningan regency	0.05	0.08	0.10	0.26	0.30	0.34	0.35	0.46	0.58	0.43	0.67	0.42	0.25	0.37	0.43	0.49	0.68	0.70	0.44	0.24
*9*	Cirebon regency	0.02	0.03	0.04	0.10	0.12	0.14	0.14	0.19	0.24	0.18	0.28	0.18	0.12	0.19	0.21	0.22	0.28	0.28	0.17	0.09
*10*	Majalengka regency	0.01	0.02	0.03	0.08	0.10	0.11	0.11	0.15	0.18	0.13	0.21	0.12	0.07	0.11	0.12	0.13	0.17	0.17	0.11	0.06
*11*	Sumedang regency	0.03	0.05	0.06	0.17	0.19	0.23	0.23	0.30	0.37	0.26	0.38	0.22	0.13	0.18	0.20	0.20	0.25	0.25	0.15	0.08
*12*	Indramayu regency	0.03	0.04	0.06	0.16	0.19	0.24	0.25	0.36	0.48	0.37	0.58	0.38	0.24	0.37	0.42	0.46	0.64	0.63	0.40	0.21
*13*	Subang regency	0.05	0.09	0.11	0.33	0.41	0.54	0.60	0.81	1.03	0.75	1.12	0.69	0.43	0.63	0.66	0.65	0.81	0.81	0.51	0.28
*14*	Purwakarta regency	0.08	0.13	0.17	0.46	0.53	0.63	0.63	0.84	1.02	0.70	1.01	0.59	0.34	0.48	0.53	0.58	0.77	0.81	0.52	0.28
*15*	Karawang regency	0.03	0.05	0.07	0.20	0.21	0.24	0.23	0.30	0.38	0.28	0.45	0.31	0.19	0.29	0.35	0.40	0.53	0.54	0.34	0.18
*16*	Bekasi regency	0.13	0.21	0.27	0.77	0.88	1.03	1.05	1.40	1.78	1.34	2.09	1.35	0.84	1.10	1.13	1.08	1.29	1.25	0.79	0.42
*17*	Bandung Barat regency	0.09	0.14	0.18	0.53	0.65	0.76	0.73	0.95	1.16	0.81	1.19	0.72	0.43	0.63	0.74	0.81	1.04	1.06	0.67	0.36
*18*	Pangandaran regency	0.05	0.08	0.10	0.28	0.33	0.40	0.43	0.59	0.77	0.59	0.96	0.65	0.43	0.69	0.88	1.00	1.39	1.51	0.96	0.51
*19*	Bogor city	0.36	0.61	0.79	2.25	2.50	2.74	2.58	3.26	3.91	2.78	4.17	2.61	1.61	2.33	2.67	2.85	3.76	3.91	2.51	1.34
*20*	Sukabumi city	0.32	0.52	0.68	2.01	2.54	3.27	3.41	4.35	5.11	3.42	4.78	2.78	1.65	2.37	2.73	3.05	4.25	4.23	2.66	1.42
*21*	Bandung city	0.34	0.56	0.75	2.19	2.68	3.10	2.98	3.73	4.33	2.92	4.15	2.47	1.42	1.94	2.04	2.05	2.55	2.52	1.60	0.86
*22*	Cirebon city	0.08	0.14	0.17	0.49	0.59	0.73	0.77	1.07	1.38	1.06	1.74	1.13	0.74	1.16	1.44	1.71	2.55	2.90	1.85	0.99
*23*	Bekasi city	0.29	0.47	0.60	1.69	1.96	2.39	2.58	3.74	5.13	3.70	5.55	3.22	1.86	2.58	2.86	3.02	3.92	3.94	2.52	1.35
*24*	Depok city	0.46	0.76	1.00	2.98	3.66	4.64	4.90	6.60	8.27	6.15	9.79	6.45	4.14	5.93	6.61	6.92	8.86	8.70	5.50	2.94
*25*	Cimahi city	0.31	0.52	0.69	2.04	2.50	3.06	3.07	3.99	4.69	3.19	4.52	2.71	1.53	2.10	2.29	2.43	3.19	3.24	2.06	1.10
*26*	Tasikmalaya city	0.08	0.13	0.17	0.46	0.55	0.67	0.70	0.93	1.13	0.77	1.06	0.61	0.33	0.43	0.46	0.46	0.58	0.59	0.37	0.20
*27*	Banjar city	0.09	0.15	0.19	0.53	0.63	0.75	0.75	0.98	1.20	0.86	1.32	0.78	0.45	0.63	0.70	0.71	0.90	0.90	0.56	0.30

^a^ The estimated and predicted values are obtained as: Relative risk ${\hat{\theta }}_{\mathrm{it}}=\text{exp}({\hat{\eta }}_{\mathrm{it}})$.

**Table B7:** Estimated (Weeks 1–18) and predicted (Weeks 19–20) posterior exceedance probability of the relative risk by county

		Week																			
*ids*	County	1	2	3	4	5	6	7	8	9	10	11	12	13	14	15	16	17	18	19	20
*1*	Bogor regency	0.00	0.00	0.00	0.03	0.07	0.16	0.17	0.43	0.68	0.35	0.87	0.37	0.04	0.26	0.34	0.35	0.58	0.54	0.21	0.04
*2*	Sukabumi regency	0.00	0.00	0.00	0.00	0.00	0.00	0.00	0.00	0.00	0.00	0.00	0.00	0.00	0.00	0.00	0.00	0.00	0.00	0.00	0.00
*3*	Cianjur regency	0.00	0.00	0.00	0.00	0.00	0.00	0.00	0.00	0.00	0.00	0.00	0.00	0.00	0.00	0.00	0.00	0.00	0.00	0.00	0.00
*4*	Bandung regency	0.00	0.00	0.00	0.00	0.01	0.03	0.03	0.12	0.25	0.05	0.27	0.03	0.00	0.02	0.04	0.07	0.24	0.26	0.10	0.02
*5*	Garut regency	0.00	0.00	0.00	0.00	0.00	0.00	0.00	0.00	0.00	0.00	0.00	0.00	0.00	0.00	0.00	0.00	0.00	0.00	0.00	0.00
*6*	Tasikmalaya regency	0.00	0.00	0.00	0.00	0.00	0.00	0.00	0.00	0.00	0.00	0.00	0.00	0.00	0.00	0.00	0.00	0.00	0.00	0.00	0.00
*7*	Ciamis regency	0.00	0.00	0.00	0.00	0.00	0.00	0.00	0.00	0.00	0.00	0.01	0.00	0.00	0.00	0.00	0.00	0.02	0.03	0.02	0.00
*8*	Kuningan regency	0.00	0.00	0.00	0.00	0.00	0.00	0.00	0.02	0.06	0.01	0.10	0.01	0.00	0.00	0.01	0.02	0.12	0.14	0.06	0.01
*9*	Cirebon regency	0.00	0.00	0.00	0.00	0.00	0.00	0.00	0.00	0.00	0.00	0.00	0.00	0.00	0.00	0.00	0.00	0.00	0.00	0.00	0.00
*10*	Majalengka regency	0.00	0.00	0.00	0.00	0.00	0.00	0.00	0.00	0.00	0.00	0.00	0.00	0.00	0.00	0.00	0.00	0.00	0.00	0.00	0.00
*11*	Sumedang regency	0.00	0.00	0.00	0.00	0.00	0.00	0.00	0.00	0.00	0.00	0.00	0.00	0.00	0.00	0.00	0.00	0.00	0.00	0.00	0.00
*12*	Indramayu regency	0.00	0.00	0.00	0.00	0.00	0.00	0.00	0.00	0.02	0.00	0.05	0.00	0.00	0.00	0.01	0.02	0.08	0.10	0.04	0.01
*13*	Subang regency	0.00	0.00	0.00	0.00	0.01	0.03	0.05	0.21	0.47	0.16	0.55	0.11	0.01	0.06	0.10	0.10	0.23	0.24	0.09	0.01
*14*	Purwakarta regency	0.00	0.00	0.00	0.02	0.04	0.08	0.08	0.25	0.45	0.12	0.43	0.06	0.00	0.02	0.04	0.06	0.20	0.24	0.09	0.01
*15*	Karawang regency	0.00	0.00	0.00	0.00	0.00	0.00	0.00	0.00	0.00	0.00	0.01	0.00	0.00	0.00	0.00	0.00	0.03	0.05	0.02	0.00
*16*	Bekasi regency	0.00	0.00	0.00	0.17	0.29	0.46	0.49	0.79	0.94	0.76	0.99	0.78	0.22	0.53	0.56	0.49	0.64	0.59	0.24	0.06
*17*	Bandung Barat regency	0.00	0.00	0.00	0.04	0.08	0.16	0.15	0.37	0.60	0.22	0.62	0.14	0.01	0.08	0.15	0.21	0.45	0.46	0.17	0.03
*18*	Pangandaran regency	0.00	0.00	0.00	0.00	0.01	0.02	0.02	0.08	0.21	0.08	0.38	0.11	0.01	0.14	0.30	0.42	0.71	0.78	0.34	0.09
*19*	Bogor city	0.01	0.08	0.21	0.99	1.00	1.00	0.99	1.00	1.00	1.00	1.00	1.00	0.89	0.99	1.00	1.00	1.00	1.00	0.87	0.53
*20*	Sukabumi city	0.01	0.06	0.14	0.93	0.99	1.00	1.00	1.00	1.00	1.00	1.00	0.99	0.86	0.98	0.99	1.00	1.00	1.00	0.87	0.56
*21*	Bandung city	0.01	0.06	0.17	0.98	1.00	1.00	1.00	1.00	1.00	1.00	1.00	1.00	0.79	0.97	0.96	0.96	0.99	0.98	0.64	0.28
*22*	Cirebon city	0.00	0.00	0.00	0.05	0.09	0.18	0.21	0.48	0.72	0.47	0.89	0.54	0.17	0.57	0.76	0.88	0.99	1.00	0.74	0.36
*23*	Bekasi city	0.00	0.02	0.07	0.90	0.95	0.99	1.00	1.00	1.00	1.00	1.00	1.00	0.95	1.00	1.00	1.00	1.00	1.00	0.86	0.53
*24*	Depok city	0.03	0.19	0.41	1.00	1.00	1.00	1.00	1.00	1.00	1.00	1.00	1.00	1.00	1.00	1.00	1.00	1.00	1.00	0.99	0.88
*25*	Cimahi city	0.01	0.05	0.14	0.96	0.99	1.00	1.00	1.00	1.00	1.00	1.00	1.00	0.83	0.97	0.98	0.98	1.00	1.00	0.78	0.41
*26*	Tasikmalaya city	0.00	0.00	0.00	0.03	0.05	0.11	0.13	0.34	0.57	0.18	0.48	0.07	0.00	0.02	0.02	0.03	0.09	0.09	0.04	0.00
*27*	Banjar city	0.00	0.00	0.00	0.08	0.13	0.21	0.20	0.40	0.57	0.29	0.66	0.22	0.03	0.12	0.17	0.18	0.32	0.32	0.12	0.03
